# NIR-II AIEgens for Phototheranostics: Design, Applications and Perspectives

**DOI:** 10.3390/bios16040219

**Published:** 2026-04-14

**Authors:** Baoqing Zhao, Xianchuan Zeng, Yuyao Su, Kui Ren, Zhijun Zhang, Fei Zhang, Dong Wang

**Affiliations:** 1 School of Pharmacy, Hubei University of Science and Technology, Xianning 437100, China; baoqingzhao@hbust.edu.cn (B.Z.); zengxianchuan@hbust.edu.cn (X.Z.); 2 Center for AIE Research, Shenzhen Key Laboratory of Polymer Science and Technology, Guangdong Research Center for Interfacial Engineering of Functional Materials, College of Materials Science and Engineering, Shenzhen University, Shenzhen 518060, China; 2022200033@email.szu.edu.cn (Y.S.); kuiren@whu.edu.cn (K.R.); zhijunzhang@szu.edu.cn (Z.Z.)

**Keywords:** second near-infrared (NIR-II), fluorescence imaging (FLI), photoacoustic imaging (PAI), photothermal therapy (PTT), photodynamic therapy (PDT), aggregation-induced emission (AIE)

## Abstract

The design of novel aggregation-induced emission (AIE)-active molecules represents a cutting-edge strategy for integrated phototheranostics in the second near-infrared (NIR-II) window. This review systematically outlines rational molecular engineering approaches based on D-A, D-A-D, and A-D-A systems to achieve red-shifted NIR-II absorption/emission, enhanced AIE characteristics, and balanced radiative and non-radiative decay pathways. These AIEgens enable high-contrast NIR-II fluorescence imaging (FLI) and photoacoustic imaging (PAI) for precise tumor localization, while concurrently facilitating efficient photothermal therapy (PTT) and robust photodynamic therapy (PDT) through both type-I and type-II mechanisms. Nanoformulations of these molecules exhibit excellent stability, biocompatibility, and passive targeting via the enhanced permeability and retention (EPR) effect. We further highlight representative “all-in-one” AIE platforms that demonstrate synergistic PTT/PDT under multimodal imaging guidance, offering a promising paradigm for precision cancer theranostics. Challenges and future directions in clinical translation and combination therapy are also discussed.

## 1. Introduction

Malignant tumors are the leading cause of death worldwide, which has prompted researchers to explore more precise and efficient comprehensive treatment approaches. Traditional cancer treatment methods, such as chemotherapy and radiotherapy, often have problems like strong systemic toxicity, poor selectivity, and susceptibility to drug resistance [1,2]. In recent years, optical medical technology has integrated diagnostic imaging with photodynamic therapy, presenting broad application prospects. This technology offers precise temporal and spatial controllability, minimally invasive characteristics, and the ability to monitor therapeutic efficacy in real time, thus attracting much attention [3,4,5,6,7,8]. Specifically, the study found that compared to the first near-infrared (NIR-I) spectral window (700–900 nm), the NIR-II (1000–1700 nm) FLI can achieve approximately 3–5 times greater penetration depth in tissues (up to 1–2 cm, while the NIR-I is 0.5–1 cm). This is because the scattering and absorption of photons by tissue components (such as water and lipids) are reduced. Additionally, NIR-II FLI typically shows 1–2 orders of magnitude lower tissue autofluorescence, thereby significantly increasing the signal-to-background ratio (usually above 10–100, while the NIR-I is below 5–10) [9,10,11,12]. These characteristics make NIR-II probes exhibit unique advantages in precise tumor identification, vascular visualization, and image-guided interventional procedures [13,14]. Additionally, the light absorption characteristics of the NIR-II band also make it suitable for photothermal therapy and photodynamic therapy. The former converts light energy into heat for tumor ablation, while the latter generates cytotoxic reactive oxygen species (ROS) to induce cell death. How to integrate the fluorescence enhancement effect, light absorption enhancement effect, photothermal therapy, and photodynamic therapy into a single multifunctional platform is an attractive yet challenging goal in the field of nanomedicine [15,16,17].

One of the main difficulties in building such an “integrated” system lies in the fact that the aggregation-induced emission effect is widespread. Traditional fluorescent markers and photosensitizers often experience fluorescence quenching and reduced efficiency of ROS generation when they are in an aggregated state—that is, the state they present in the biological environment or when they are formulated into nanoparticles for delivery. This limitation severely restricts the imaging brightness and therapeutic effect.

The discovery and development of aggregation-induced emission (AIE) materials provide a powerful solution to this challenge. AIE materials emit weakly in the molecular dissolved state, but become highly luminescent when aggregated due to restricted intramolecular motion (RIM) [18,19,20,21,22]. This unique “onset” property not only avoids the ACQ effect but also provides a multifunctional platform for regulating the excitation energy path. Through rational molecular design, the radiative decay path (for fluorescence and photo-induced ionization) and the non-radiative decay path (for heat generation in photothermal therapy and intersystem crossing in photodynamic therapy) can be finely regulated [23].

**Scheme 1:**
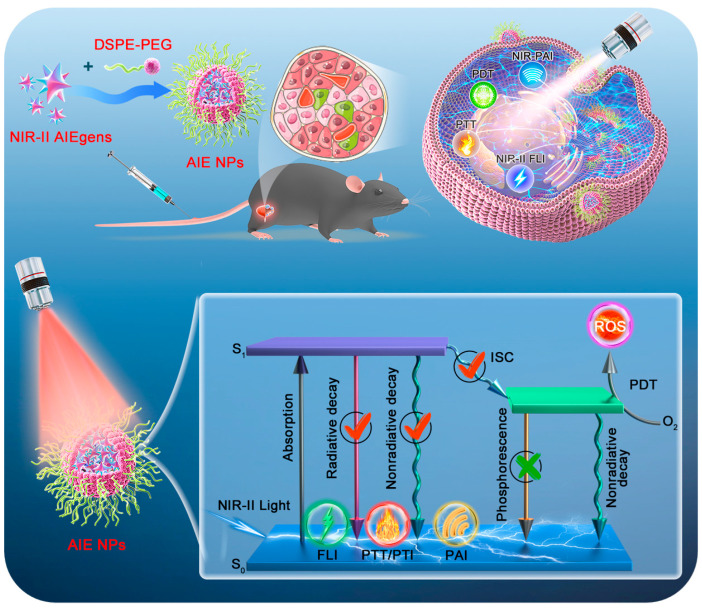
Schematic depiction of the integrated application pipeline of NIR-II AIE nanoparticles for multimodal imaging-guided synergistic therapy and their core operational mechanism.

AIE molecules constructed based on the donor–acceptor (D-A) structural framework have demonstrated unique advantages in this field [2,24,25,26,27]. By connecting strong electron donors (D) and acceptor (A) units through a π-conjugated bridge, this design promotes a strong intramolecular charge transfer (ICT) effect, effectively narrowing the molecular band gap and shifting the absorption and emission to the NIR-II window. Additionally, the D-A structure has high tunability: the electron-pulling and electron-pushing strength of the D/A units, the structure and length of the π bridge, as well as the introduction of large volume or twisted groups can all be systematically controlled to optimize optical properties, enhance AIE characteristics, and regulate the energy distribution of the excited state. This molecular engineering flexibility has given rise to various D-A-configured NIR-II AIEgens, including D-π-A, symmetric D-A-D, and A-D-A systems, each with unique photophysical advantages. The design core follows the same paradigm—by reasonably regulating the structure of D-A type molecules, achieving integrated NIR-II optical diagnosis and therapy functions. The common goal is to red shift the ICT effect to the NIR-II band; at the same time, by introducing twisted conformations, large volume substituents, or AIE active units, the excitation state energy decay path can be regulated, thereby balancing radiation decay (enhancing NIR-II fluorescence imaging and photoacoustic imaging) and non-radiation decay (improving photothermal/photodynamic treatment efficiency). Finally, through nanocarrier technology, a stable and biocompatible “integrated” platform is constructed to achieve multimodal imaging-guided synergistic photothermal/photodynamic therapy (Scheme 1).

Although several review articles have comprehensively summarized the NIR-II region fluorescent dyes used in biological imaging and phototherapy, most of these reviews either focus on material screening or the results of in vivo applications [28,29,30,31,32,33,34,35]. In contrast, this review aims to systematically review the rational design strategies adopted for developing D-A type NIR-II AIEgens and to deeply explore their crucial role in promoting the development of integrated optical diagnosis and therapy. We focus on analyzing how specific molecular designs achieve outstanding performance in multimodal NIR-II fluorescence/photoacoustic imaging (FLI/PAI), efficient photothermal therapy (PTT), and effective photodynamic therapy (PDT), covering both type-I and type-II mechanisms. Additionally, the discussion will also cover these AIEgens’ nanodelivery systems, highlighting their advantages in improving biocompatibility, stability, and tumor targeting ability. By analyzing breakthrough cases in the recent literature, this article will emphasize how the integration of NIR-II optics, AIE phenomena, and D-A molecular engineering gives rise to a new generation of intelligent, multifunctional optical diagnosis and therapy platforms. Finally, we summarize the current challenges and future prospects of this active field, and point out its broad prospects in clinical translation and next-generation combined treatment strategies.

This narrative review aims to summarize the rational molecular design strategies for NIR-II AIE materials used for integrated optical diagnosis and treatment. Here, the NIR-II capability is defined as the emission wavelength extending to the range of 1000–1500 nm. Whether the maximum emission is within this range or not, as long as it can achieve practical NIR-II fluorescence imaging, it is acceptable. By searching for the following keyword combinations in the Web of Science database: “NIR-II “, “AIE”, “FLI”, “PAI”, “PTT”, and “PDT”, the relevant literature was identified. The search time range was from 2015 to 2025, with a focus on significant progress reported between 2020 and 2025. The review mainly covers the material’s photophysical characterization, quantitative therapeutic performance, and in vivo multimodal imaging and its therapeutic research. It further clarifies the key design principles in D-A, D-A-D, and A-D-A molecular architectures.

## 2. Rational Design of NIR-II AIEgens for “All-in-One” Phototheranostics

At present, the design strategies and advantages of NIR-II AIE materials for integrating fluorescence/photoacoustic imaging (FLI/PAI), photodynamic therapy (PDT), and photothermal therapy (PTT) can be summarized as follows. The rational molecular design adopts D-A structures (D-A, D-A-D, A-D-A), in which the donors/acceptors are connected together through conjugated π bridges. This enhances the coupling efficiency, narrows the band gap, and shifts the emission wavelength to the NIR-II region. By twisting and large molecular clusters to suppress π-π stacking, ACQ is transformed into AIE. This design precisely balances the energy dissipation of the excited state: radiative decay is used for high-brightness FLI/PAI, and non-radiative decay is used for efficient photothermal conversion (PTT) and ROS generation (PDT).

The obtained NIR-II AIEgens offer significant advantages for multimodal optical diagnosis and treatment: strong NIR-II absorption and AIE-enhanced fluorescence can achieve high-sensitivity FLI, while also having high-resolution PAI, which is suitable for precise tumor localization. The absorbed light energy is efficiently directed along non-radiative paths, achieving a high photothermal conversion efficiency and enabling efficient PTT. Through molecular engineering, ROS generation can also be promoted, achieving potent PDT (including hypoxia-tolerant type-I PDT).

The diagnosis (FLI/PAI) and treatment (PTT/PDT) are integrated into a single molecule or its nanoformulation, forming an “integrated” platform. After being encapsulated by amphiphilic polymers, the photosensitizer exhibits excellent water dispersibility and passive tumor targeting ability (EPR effect). In vivo, this platform can achieve clear imaging guidance and synergistic PTT/PDT, achieving complete ablation without recurrence in various tumor models. Therefore, the rational design of D-A type NIR-II AIEgens provides a powerful strategy for precise multimodal imaging and efficient combined treatment, promoting the development of image-guided integrated cancer diagnosis and treatment.

### 2.1. Design Strategies of D-A Type Molecules and Their Advantages in NIR-II FLI/PAI, PTT/PDT, and AIE Systems

The rational construction of D-A type molecules is the key to developing advanced AIE fluorescent materials for next-generation optical therapy and diagnostic technologies (especially those operating in the NIR-II window). The core idea is to connect the strong electron donor and the strong electron acceptor in a directional manner through a conjugated π bridge, forming a D-π-A structure. By regulating the intramolecular charge transfer (ICT) process, precise control over the optical properties can be achieved. In terms of specific design strategies, first, the donor should have a strong electron-donating ability, such as using triphenylamine (TPA) groups with a non-planar helical structure, which can effectively inhibit unfavorable π-π stacking in the aggregated state, thereby alleviating aggregation-induced quenching (ACQ) and enhancing AIE effect [36,37,38]. Secondly, the acceptor should have a strong electron-withdrawing ability, such as methylated acridine or benzothiazole derivatives (e.g., 2-methyl-1-(3-sulfonic acid propyl)-benzothiazole), and through structural modification, its electron-withdrawing strength can be further enhanced, thereby effectively reducing the energy gap [25,27,39]. Furthermore, by introducing heterocyclic units such as thiophene to extend the π conjugated bridge, the number of thiophene units can be increased to elongate the conjugation length, causing the absorption and emission spectra to red shift to the NIR-II region; at the same time, these thiophene rings act as “molecular rotors” in solution, which can promote non-radiative energy dissipation through free rotation, while their rotation is restricted in the aggregated state, thereby activating the radiative decay channel and generating strong emission. This fine regulation of molecular motion is the core for achieving a balance between the radiative (used for fluorescence imaging and photo-activated imaging) and non-radiative (used for photothermal therapy) decay channels in a single excited state [24,40]. Finally, introducing functional groups enhances the targeting ability. For instance, introducing hydrophilic sulfonic acid groups can form amphiphilic molecules, which can self-assemble into nanoparticles in water, thereby improving water solubility and cell-specific targeting performance [23,41].

**Scheme 2:**
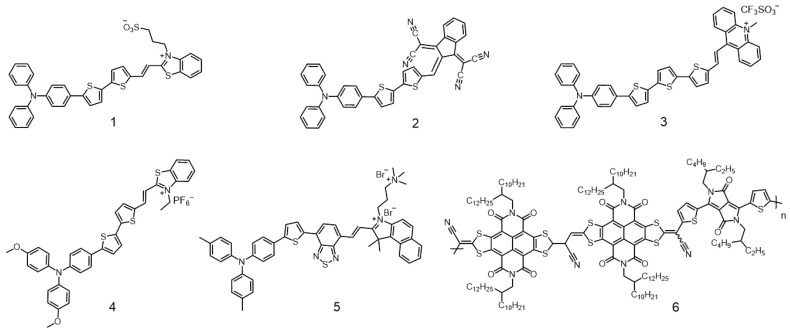
Molecular structures of D-A-structured AIE compounds for NIR-II FLI, PAI, and applications in PTT and PDT.

Traditional organic fluorescent markers and photosensitizers for in vivo phototherapy diagnostic techniques encounter two major problems: one is the ACQ effect, which significantly reduces the fluorescence brightness and the efficiency of ROS generation in nanoparticle formulations; the other is the limited tissue penetration ability of visible light/NIR-I light [42,43,44,45]. A series of studies (2020–2025) demonstrates a rational molecular design strategy (e.g., TDTMSB (compound **1**) [41], TSSI (compound **2**) [23], TSSAM (compound **3**) [46], SP3 (compound **4**) [47], TTT-4 (compound **5**) [48], CyQN-BTT (compound **6**) [49]) centered on phototheranostics (Scheme 2). By systematically engineering strong D-A cores featuring electron-donating units and electron-accepting units, and extending the π-conjugated bridge, the researchers precisely tune photophysical properties. This D-A structural optimization effectively red shifts absorption and emission into the NIR-II window, narrows energy gaps, enhances AIE characteristics, and boosts both ROS generation and photothermal conversion. For example, systematic extension of the π-conjugated bridge from a single phenyl linker (TMSB, λ_abs_ = 525 nm) to a bithiophene-phenyl system (TDTMSB, λ_abs_ = 560 nm, λ_em_ = 700–1100 nm) results in a 35 nm red shift in absorption and extends emission into the NIR-II region, enabling deeper tissue imaging. The resulting amphipathic molecules form stable nanoparticles, functioning as “all-in-one” platforms. They enable high-contrast NIR-II FLI/PAI and synergize PTT with type I/II PDT for precise and potent tumor eradication.

**Figure 1:**
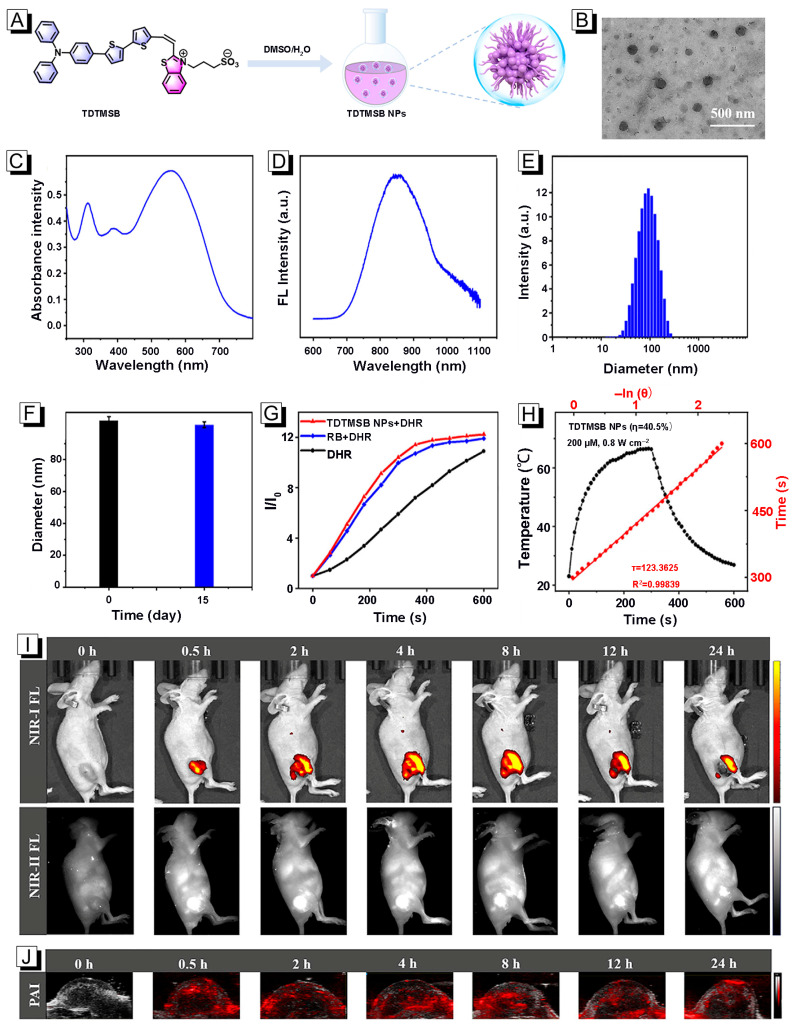
(**A**) Self-assembly of TDTMSB NPs. (**B**) TEM image (scale bar: 500 nm). (**C**) Absorption and (**D**) fluorescence emission spectra. (**E**) Hydrodynamic size distribution. (**F**) Stability over 15 days at 4 °C. (**G**) •O_2_^−^ detection using DHR123. (**H**) Temperature rise under 635 nm irradiation. (**I**) In vivo NIR-I/II FLI time series. (**J**) Corresponding in vivo PAI. Reprinted from ref. [41].

In 2025, Ma and others reported a new type of amphiphilic TDTMSB (Compound **1**) [41]. TDTMSB, featured with D-A interaction and extended π-conjugation, this AIE-active molecule overcomes ACQ and exhibits an absorption peak at 560 nm and long-wavelength emission (~865 nm) extending into the NIR-II region (Figure 1C,D). The high-sensitivity NIR-II FLI and complementary high-resolution PAI are shown in tumor-bearing mice (Figure 1I,J). To facilitate biological application, TDTMSB self-assembles into stable, uniformly sized nanoparticles with a diameter of ~100 nm (Figure 1A,B,E,F). These NPs achieve a high photothermal conversion efficiency of 40.5% for effective PTT (Figure 1H). Critically, they also generate multiple ROS, including superoxide radical (•O_2_^−^, Figure 1G). This study reports a bi-functional AIE phototherapy diagnostic agent, which successfully achieved multimodal NIR-II fluorescence/photothermal multimodal imaging-guided synergistic type I/II photodynamic and photothermal therapy.

**Figure 2:**
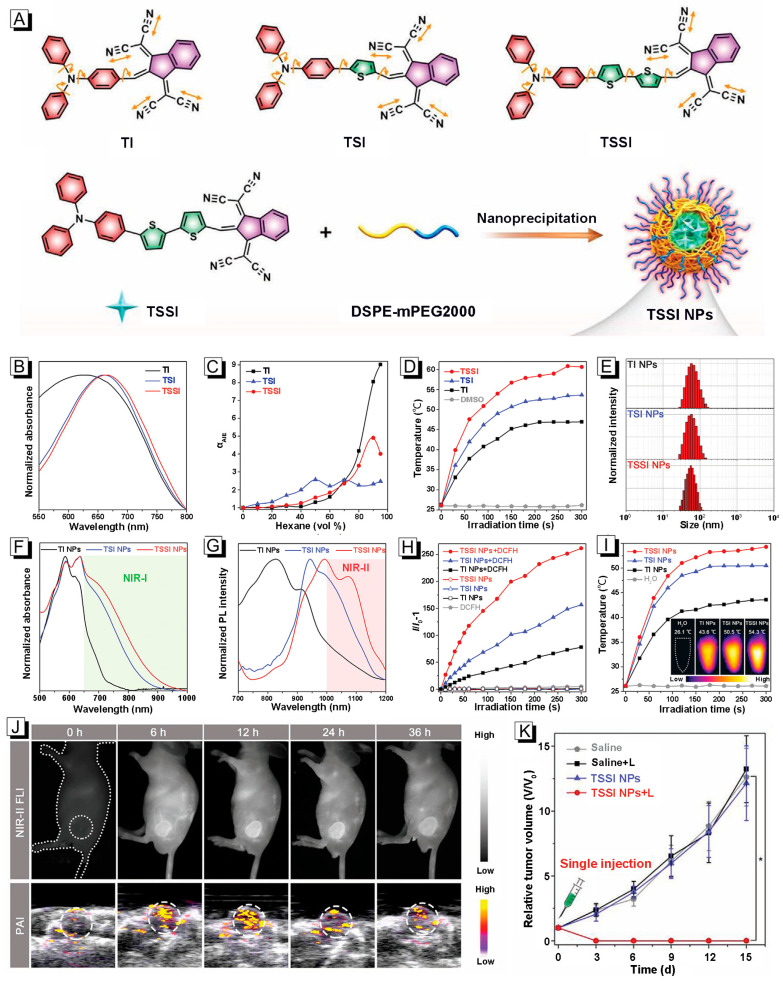
(**A**) Molecular structures of TI, TSI, TSSI and NP preparation. (**B**) Solid-state absorption spectra. (**C**) AIE behavior with hexane fraction. (**D**) Photothermal heating in DMSO under a 660 nm laser. (**E**) NP size distribution. (**F**,**G**) Normalized absorption and emission spectra in water. (**H**) ROS generation under a laser. (**I**) Photothermal performance in aqueous solution. (**J**) In vivo NIR-II FLI and PAI of tumors. (**K**) Tumor growth kinetics (* *p* < 0.05. Student’s *t*-test). Reprinted with permission from ref. [23]. © John Wiley and Sons (2020).

In 2021, Zhang et al. present a novel and integrative phototheranostic strategy by designing a single-molecule, AIE-active fluorophore TSSI (compound **2**) that simultaneously enables NIR-II FLI, PAI, PTI, PDT, and PTT [23]. As illustrated in Figure 2A, the molecular architecture is ingeniously constructed with a strong electron D-A core, featuring a 1,3-bis(dicyanomethylidene)indane acceptor, a triphenylamine donor, and a thiophene bridge. This design capitalizes on the inherent molecular rotors and vibrators of AIEgens to finely modulate the balance between radiative decay (for fluorescence) and non-radiative decay (for heat, Figure 2D) and ROS generation). The hydrophobic TSSI is subsequently encapsulated into uniform, biocompatible nanoparticles using the amphiphilic copolymer DSPE-mPEG2000 via a straightforward nanoprecipitation method (Figure 2A), yielding TSSI NPs with an average size of ~55.5 nm (Figure 2E), ideal for tumor accumulation via the EPR effect. TSSI and TSSI NPs exhibit a broad absorption spectrum in the NIR region (Figure 2B,F), enabling deep-tissue activation. They exhibit NIR-II fluorescence emission (Figure 2G), which is directly attributed to their AIE properties (Figure 2C). Under 660 nm laser irradiation, TSSI nanoparticles demonstrate dual capabilities: they can generate ROS (Figure 2H) and have a high photothermal conversion efficiency (Figure 2I). The in vivo efficacy of this system is demonstrated in Figure 2J,K. After administration, TSSI nanoparticles efficiently accumulate in 4T1 tumors (Figure 2J). After a single laser irradiation at 12 h post-injection, the tumor can be completely eliminated and does not recur (Figure 2K), highlighting a powerful and controllable therapeutic effect.

**Figure 3:**
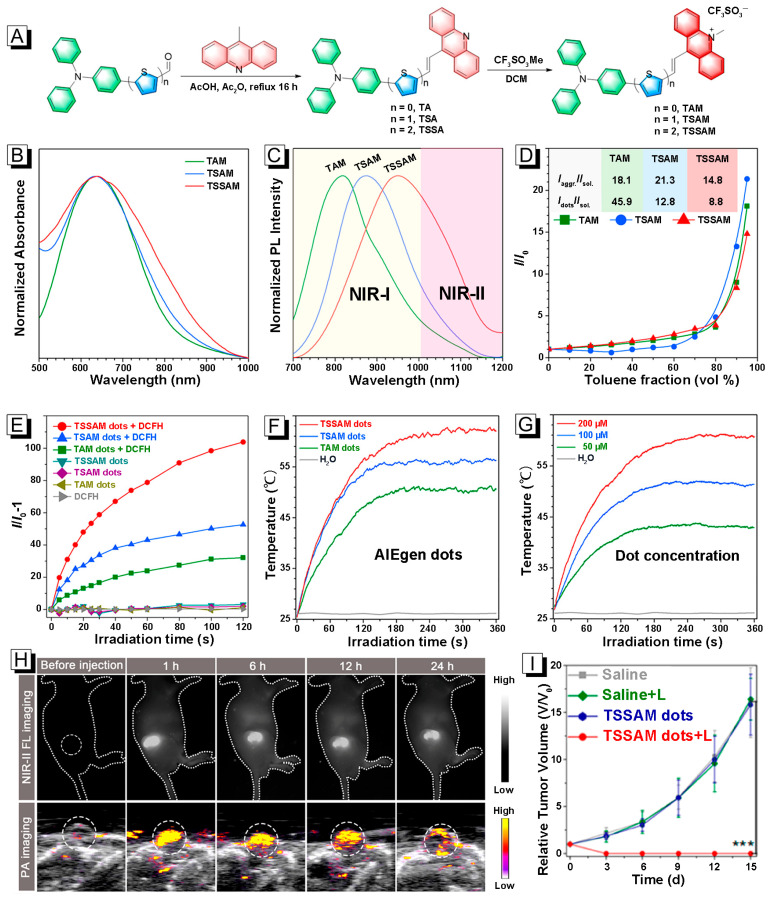
(**A**) Synthesis of TAM, TSAM, TSSAM. (**B**) Solid-state absorption profiles. (**C**) Emission spectra in aggregates. (**D**) AIE behavior in DMSO/toluene. (**E**) ROS generation of AIE dots. (**F**,**G**) Photothermal performance. (**H**) In vivo NIR-II FLI and PAI after intratumoral injection. (**I**) Tumor volume progression (*** *p* < 0.001 represent statistically significant difference). Reprinted with permission from ref. [46]. © American Chemical Society (2020).

In 2020, Xu et al. reported a novel phototheranostic engineering AIE-active single-molecule system, TSSAM (compound **3**) [46]. As illustrated in Figure 3A, TSSAM was synthesized along with analogs TAM and TSAM, featuring a D-A structure. The absorption (Figure 3B) showed broad coverage extending to 1000 nm, and fluorescence spectra (Figure 3C) in the NIR-II window with AIE characteristic (Figure 3D). In functional assessments, Figure 3E reveals the ROS generation ability and the photothermal performance shown in Figure 3F,G. After intratumoral injection of TSSAM points at the tumor site, strong NIR-II region fluorescence and photoacoustic signals were observed (Figure 3H). When combined with a single laser irradiation, TSSAM points can completely eliminate 4T1 tumors without recurrence, demonstrating the synergistic effect of PDT/PTT (Figure 3I).

**Figure 4:**
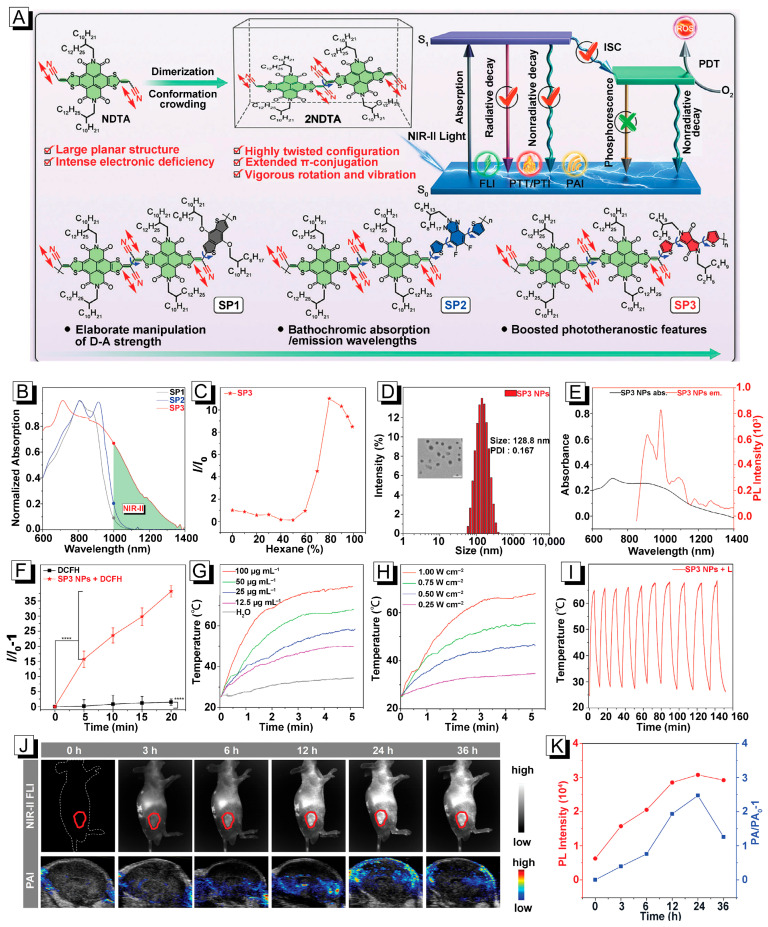
(**A**) Molecular design of SP3 for imaging-guided phototherapy. (**B**) Absorption spectra in THF. (**C**) AIE behavior in CHCl_3_/hexane. (**D**) Size and TEM of SP3 NPs. (**E**) Absorption/emission in water. (**F**) ROS generation under a 1064 nm laser (**** *p* < 0.0001, Student’s *t*-test). (**G**,**H**) Photothermal performance at different concentrations/powers. (**I**) Photothermal cycling stability. (**J**) In vivo NIR-II FLI and PAI post-injection. (**K**) Signal intensity at the tumor over time. Reprinted with permission from ref. [47]. © John Wiley and Sons (2024).

By designing a single-molecule semiconductor polymer SP3 (compound **4**) with AIE activity, a novel and comprehensive phototherapy diagnostic strategy was proposed [47]. As shown in Figure 4A, the molecule with 2NDTA features a highly distorted conformation for AIE. The photophysical verification is shown in Figure 4B, and its absorption range extends to the NIR-II band. After the preparation of nanoparticles, SP3 NPs exhibit strong NIR-II fluorescence (Figure 4E), and the AIE behavior was confirmed in a hexadecane/chloroform mixture (Figure 4C). These NPs have excellent colloidal stability (Figure 4D). SP3 NPs can effectively generate ROS under 1064 nm laser irradiation. This is confirmed by the enhancement of DCFH fluorescence (Figure 4F) and rapid and concentration-dependent photothermal heating (Figure 4G,H). This photothermal performance remains stable after ten heating–cooling cycles (Figure 4I). After intravenous injection into tumor-bearing mice, the NIR-II region fluorescence and photoacoustic signals at the tumor site increase over time, reaching a peak at 24 h after injection (Figure 4J,K), providing guidance for subsequent photoligated treatment.

**Figure 5:**
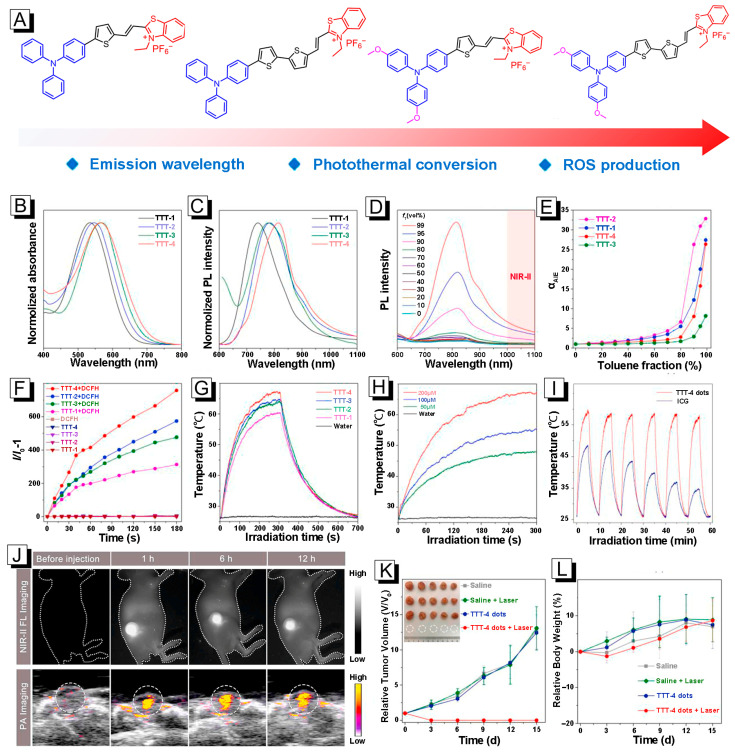
(**A**) Structures of TTT-1–TTT-4. (**B**) Absorption spectra in DMSO. (**C**,**D**) PL spectra and AIE behavior in DMSO/toluene. (**E**) Relative intensity vs. toluene fraction. (**F**) ROS generation of AIE dots. (**G**,**H**) Photothermal response. (**I**) Photothermal cycling vs. ICG. (**J**) In vivo NIR-II FLI and PAI post-injection. (**K**) Tumor growth curves. (**L**) Body weight changes. Reprinted with permission from ref. [48]. © Elsevier (2021).

In 2021, Wen et al. presented a novel TTT-4 (compound **5**) for comprehensive cancer phototheranostics [48]. The molecular design, as illustrated in Figure 5A, is strategically based on a D-A configuration. The optical properties of these compounds, detailed in Figure 5B–E, confirm their quintessential AIE behavior. They exhibit minimal emission in solution but demonstrate significantly enhanced fluorescence upon aggregation. The remarkable ROS generation capacity of TTT-4 is clearly evidenced (Figure 5F), establishing effective PDT. TTT-4 demonstrates a rapid and substantial temperature increase upon 660 nm laser irradiation, possesses a high PCE, and exhibits excellent stability (Figure 5G–I). Following intratumoral administration of TTT-4 dots, tumor delineation is achieved using NIR-II FLI and PAI (Figure 5J). This potent anti-tumor outcome is achieved without inducing significant systemic toxicity (Figure 5K,L).

**Figure 6:**
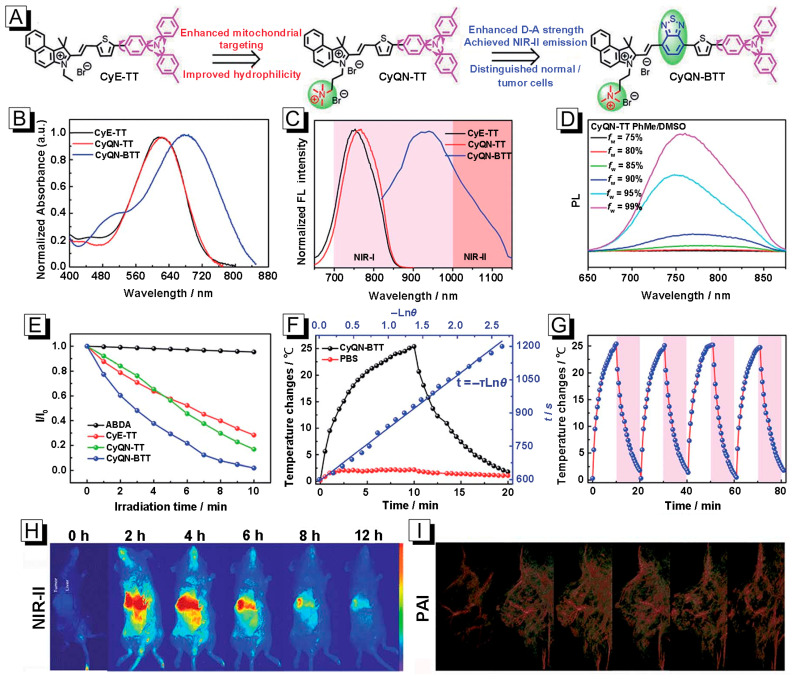
(**A**) Structures of CyE-TT, CyQN-TT, CyQN-BTT. (**B**) Absorption spectra in DMSO. (**C**) FL intensity in toluene. (**D**) FL behavior in PhMe/DMSO mixtures. (**E**) ^1^O_2_ generation using ABDA. (**F**) Photothermal curves and linear fitting. (**G**) Photothermal cycling stability. (**H**) In vivo NIR-II FLI and (**I**) PAI. Reprinted from ref. [49].

In 2023, a study was reported detailing the rational design of a single-molecule photosensitizer, CyQN-BTT (compound **6**), for multifunctional phototheranostics [49]. As illustrated in Figure 6A–C, the absorption and emission spectra extend the fluorescence tail into the NIR-II region. This NIR-II emission, along with a broad absorption profile, supports simultaneous PAI, as confirmed in vivo, where clear tumor delineation is achieved (Figure 6H,I). CyQN-BTT exhibits a unique AIE characteristic (Figure 6D). For therapy, the molecule demonstrates a high singlet oxygen yield (27.1%) for PDT and an exceptional PCE of 37.8% for PTT (Figure 6E–G).

The above compounds possess unique molecular structural frameworks, charge states, and targeting mechanisms. Compounds **2** and **4** are neutral systems: Compound **2** is a twisted D-A type small molecule with a 1,3-dichloromethylindole receptor, while Compound **4** is a semiconductor polymer in which the receptor dimerizes to induce a twisted AIE active backbone; both rely on electron spin resonance effects. Compound **3** uses a single-dialkylammoniumized acridine receptor for mitochondrial targeting. Compounds **1** and **5** are small molecules that self-assemble without the need for additional carriers: compound **1** is a zwitterionic inner salt with a sulfopropyl group on the benzothiazole group, exhibiting excellent biocompatibility and low non-specific adsorption; while compound **5** has a cationic benzothiazole group for lysosome targeting. Compound **6** is a D-A type small molecule, exhibiting inherent tumor targeting due to its structural nature. Additionally, compounds **1** to **3**, **5**, and **6** systematically adjust the π bridges (thiophene, benzothiadiazole, or double bond) to regulate the D-A strength and balance radiation/non-radiation decay. The compounds discussed in this section demonstrate several general principles for achieving integrated photothermal therapeutic functions.

### 2.2. Design Strategies of D-A-D Type Molecules and Their Advantages in NIR-II FLI/PAI, PTT/PDT, and AIE Systems

The D-A-D structural model is a core strategy for designing advanced NIR-II class AIE materials that are integrated for multimodal PTT and diagnosis [23,50,51]. This strategy involves combining a strong planar electron-accepting core (A), such as benzobis(thiadiazole) (BBTD) or its derivatives, with a twisted electron donor unit (D) (such as triphenylene), which functions as a molecular rotor [38,52]. The significant exciton-complex conversion effect in this structure effectively narrows the optical bandgap, causing the absorption to red shift towards the NIR-I region and enabling the emission to enter the NIR-II region, which is optimal for deep-tissue penetration [26]. One of the key innovations of this design lies in the utilization of the AIE phenomenon. In the solution state, the free rotation of the D unit helps with non-radiative decay, resulting in weaker fluorescence. However, in the aggregated state, RIM blocks these non-radiative pathways, thereby activating strong NIR-II photoluminescence [53,54]. This D-A-D structure has high tunability; “receptor engineering”, such as using dual receptors (D-A-A-D), can further increase the molar extinction coefficient, promote the twisting of the main chain to inhibit harmful π-π stacking, and convert the ACQ behavior into AIE [52]. At the same time, “multi-dimensional donor engineering” allows for optimizing donor strength, introducing large and hydrophobic groups to minimize solvent interactions, and carefully balancing the energy dissipation of the excited states of the radiative and non-radiative states [20,55]. The D-A-D AIE molecule integrates multiple diagnostic and therapeutic functions. Its NIR absorption property enables NIR-II FLI and PAI. The absorbed light energy is directed towards non-radiative decay for PTT, while cross-level transition can be used for PDT.

#### 2.2.1. Benzothiadiazole

**Scheme 3:**
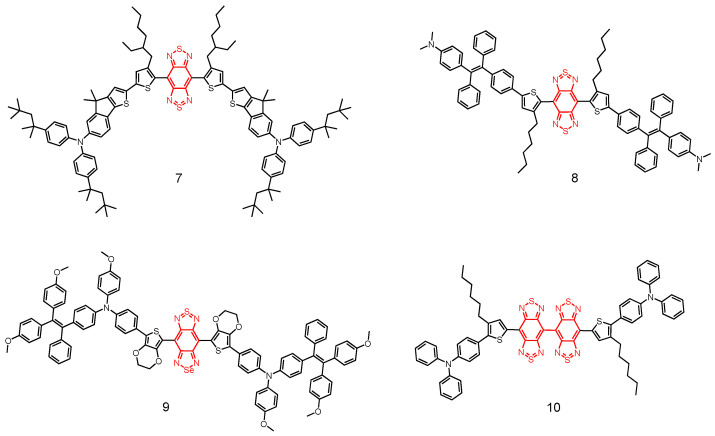
Molecular structures of D-A-D structured AIE compounds based on BTD for NIR-II fluorescence imaging, photoacoustic imaging, and applications in PTT and PDT.

The benzothiadiazole (BTD) unit serves as a fundamental and ubiquitous strong electron-accepting core in the design of NIR-II class AIE molecules. Its derivatives, such as benzodithiadiazole (BBTD) or thienylbenzothiadiazole, are widely used (Scheme 3).

**Figure 7:**
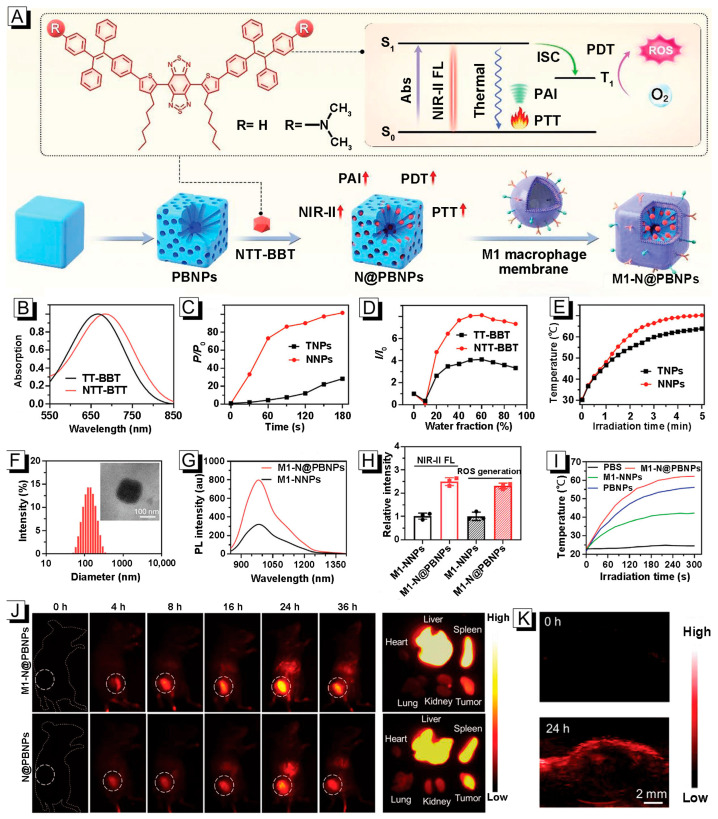
(**A**) Schematic of nanoplatform with AIEgen, mesoporous PB, and M1-membrane coating. (**B**) Absorption spectra of TT-BBT and NTT-BBT. (**C**) ROS generation under 730 nm. (**D**) AIE behavior in DMSO/water. (**E**) Photothermal heating of TNPs and NNPs. (**F**) DLS/TEM of PBNPs. (**G**) PL spectra of M1-NNPs and M1-N@PBNPs. (**H**) NIR-II fluorescence and ROS comparison. (**I**) Photothermal profiles. (**J**) In vivo NIR-II FLI after injection. (**K**) PA images pre- and post-injection. Reprinted with permission from ref. [57]. © John Wiley and Sons (2024).

In 2025, Yuan et al. reported a study on the development of OPITBT (compound **7**), a novel NIR-II AIEgen, for multimodal phototheranostics of orthotopic breast cancer [56]. This work demonstrates that multi-dimensional donor engineering can balance radiative and non-radiative energy dissipation, resulting in a high-performance “one-for-all” theranostic agent capable of NIR-II FLI, PAI, PTT, and PDT for precise cancer diagnosis and synergistic treatment. In 2024, Li et al. reported a study on a novel nanoplatform integrating a dimethylamine-substituted NIR AIEgen (NTT-BBT, compound **8**) with a mesoporous Prussian blue nanocatalyzer for enhanced cancer theranostics [57]. As illustrated in Figure 7A, the AIEgen, designed with a D-A-D structure, is encapsulated within porous Prussian blue nanoparticles (PB NPs) and further camouflaged with an M1 macrophage membrane to form the final nanoagent M1-N@PBNPs. PB NPs were fabricated through a two-step procedure: uniform cubic PB particles (≈120 nm) were synthesized hydrothermally, followed by controlled etching with HCl to generate the mesoporous structure, as verified by TEM (Figure 7F). The photoluminescence of the AIEgen is significantly enhanced in aggregate states, confirming the AIE characteristic (Figure 7D). Furthermore, the dimethylamine substitution, shown in Figure 7B, leads to a bathochromic shift in absorption, extending it into the NIR region. Critically, this modification markedly boosts both photodynamic and photothermal capabilities. The AIEgen nanoparticles (NNPs) exhibit superior ROS generation and a higher temperature increase under NIR irradiation compared to the non-aminated counterpart, highlighting the enhanced PDT and PTT potential (Figure 7C,E). Upon incorporation into the PBNPs, the molecular motion of the AIEgen is further restricted, leading to a dramatic amplification of its NIR-II fluorescence brightness and ROS production efficiency, as evidenced by the comparative data in Figure 7G,H. The porous PBNPs themselves contribute multiple functions: their catalase-like activity decomposes tumor-associated H_2_O_2_ to generate oxygen, alleviating hypoxia and further augmenting PDT. Additionally, PBNPs contribute to a strong photothermal effect, with M1-N@PBNPs showing a substantial temperature rise under laser exposure (Figure 7I). For in vivo application, the nanoplatform enables high-performance dual-modal imaging. Time-dependent NIR-II FLI of orthotopic breast tumors, with significantly higher signal from the M1 membrane-camouflaged formulation, confirming enhanced tumor targeting (Figure 7J). Complementarily, Figure 7K demonstrates robust PAI of the tumor, providing deep-tissue, high-spatial-resolution anatomical information. The integration of bright NIR-II FLI and high-contrast PAI allows for precise tumor delineation. Upon 730 nm laser irradiation at the tumor site, the combined PDT and PTT effects of the nanoagent effectively ablate cancer cells and induce immunogenic cell death, establishing a powerful platform for image-guided photoinmunotherapy. Thus, the strategic fusion of AIEgen photophysics, nanocatalytic oxygen modulation, and biomimetic targeting creates a multifaceted theranostic system for advanced cancer treatment.

**Figure 8:**
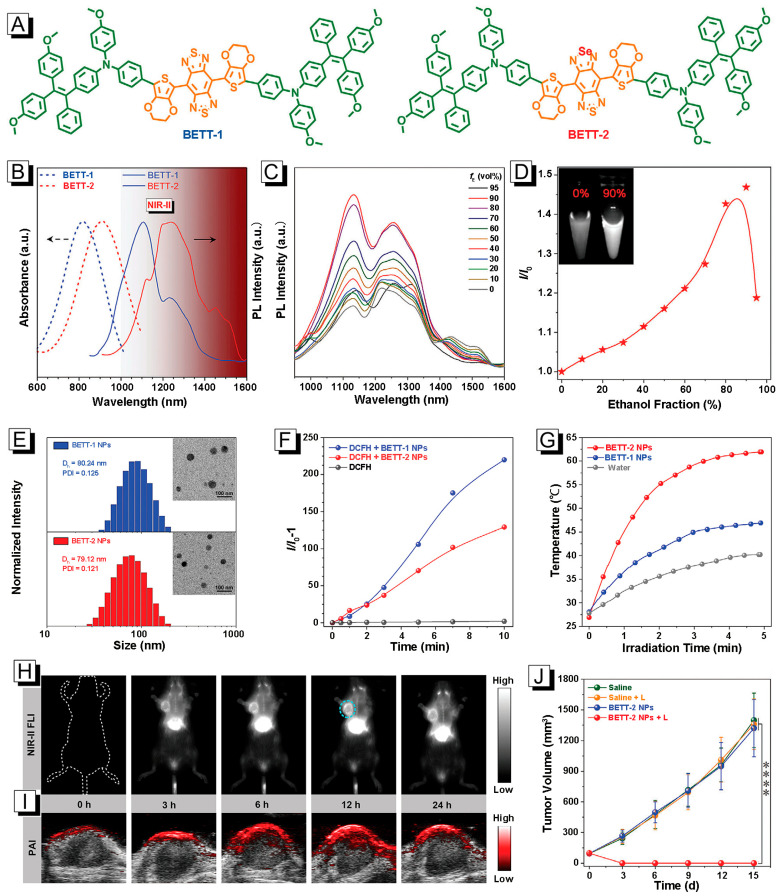
(**A**) Structures of BETT-1 and BETT-2. (**B**) Absorption/PL in CHCl_3_. (**C**) PL spectra in DMSO/ethanol. (**D**) AIE behavior. (**E**) Size and TEM of NPs. (**F**) ROS generation and (**G**) photothermal profiles under 1064 nm. (**H**) In vivo NIR-II FLI and (**I**) PAI in orthotopic model. (**J**) Tumor volume curves (**** *p* < 0.0001. Student’s *t*-test). Reprinted with permission from ref. [51]. © John Wiley and Sons (2024).

In 2024, Song et al. reported a study on a novel NIR-II excitable AIE small molecule, BETT-2 (compound **9**), designed for multimodal phototheranostics in orthotopic breast cancer treatment [51]. As illustrated in Figure 8A, BETT-2 integrates a highly twisted donor–π–acceptor–π–donor (D-π-A-π-D) architecture, combining a rotor-rich tetraphenylethylene-triphenylamine (TPE-TPA) donor, a sterically hindered 3,4-ethylenedioxythiophene (EDOT) π-bridge, and a strong electron-deficient selenadiazolo-benzo-thiadiazole (SBTD) acceptor. This molecular design simultaneously maximizes donor–acceptor strength and conformational distortion, resulting in extended absorption and emission wavelengths into the NIR-II region. Figure 8B confirms that BETT-2 exhibits a maximum absorption peak at 915 nm and an emission peak at 1240 nm in chloroform, enabling efficient excitation by a 1064 nm laser. BETT-2 exhibits typical AIE characteristics; its photoluminescence in DMSO/ethanol mixtures remains weak in pure DMSO but intensifies significantly with increasing ethanol fractions (*f_E_*), peaking at *f_E_* = 90% with bright NIR-II emission (Figure 8C,D). The subsequent intensity drop at *f_E_* = 95% is attributed to excessive aggregation and precipitation. This AIE behavior ensures strong fluorescence in the aggregated nanoparticle state, which is fundamental for its performance as an efficient NIR-II fluorescence imaging agent. After nanoformulation with DSPE-mPEG, BETT-2 NPs demonstrate excellent monodispersity with a hydrodynamic diameter of ~79 nm (Figure 8E). Under 1064 nm irradiation, these NPs exhibit robust ROS generation (Figure 8F) and a high photothermal conversion efficiency of 56.6%, leading to rapid temperature elevation (Figure 8G). In vivo, BETT-2 NPs enable high-contrast NIR-II FLI and PAI of orthotopic 4T1 breast tumors, with optimal accumulation observed at 12 h post-injection (Figure 8H,I). As shown in Figure 8J, mice treated with BETT-2 NPs plus 1064 nm laser irradiation achieve complete tumor eradication without recurrence, underscoring the potent synergistic effect of NIR-II-triggered PDT and PTT. Collectively, this work highlights the successful development of an AIE-based, single-component phototheranostic platform that integrates NIR-II FLI, PAI, PTI, PDT, and PTT, offering a promising strategy for deep-tumor imaging and non-invasive cancer therapy.

**Figure 9:**
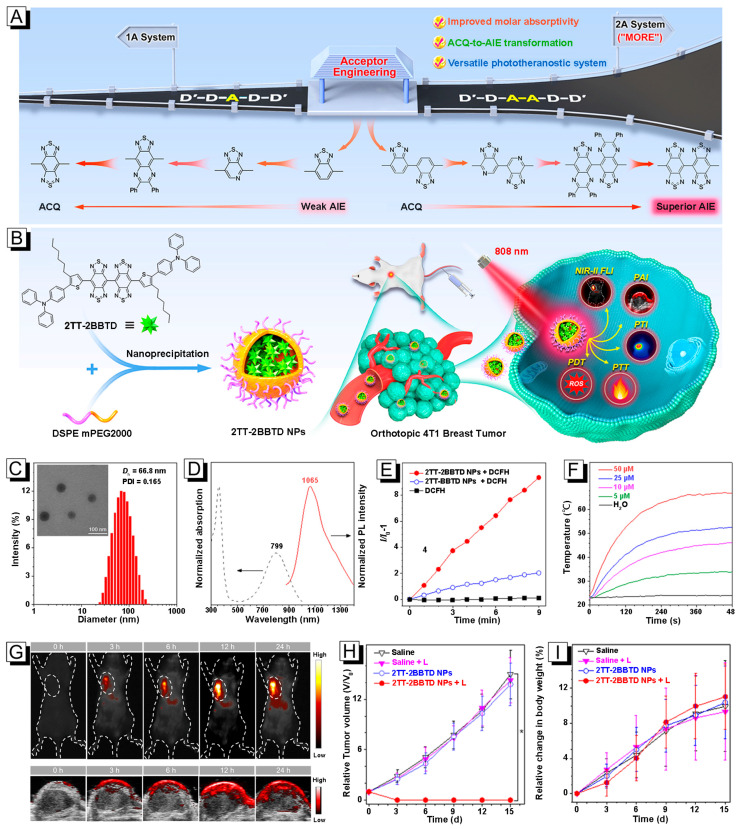
(**A**) Acceptor types for 1A/2A systems. (**B**) Structure of 2TT-2BBTD and NP preparation. (**C**) DLS/TEM of NPs. (**D**) Absorption/emission in water. (**E**) ROS generation under 808 nm. (**F**) Photothermal heating at different concentrations. (**G**) In vivo NIR-II FLI and PAI. (**H**) Tumor growth curves (* *p* < 0.05. Student’s *t*-test). (**I**) Body weight changes. Reprinted with permission from ref. [52]. © American Chemical Society (2023).

In 2023, Yang et al. reported a study on a dual-acceptor engineering strategy for constructing NIR-II AIEgens with enhanced multimodal phototheranostic capabilities [52]. As illustrated in Figure 9A, the incorporation of two electron acceptors (2A system, D′-D-A-A-D-D′) transforms ACQ into strong AIE, while the single-acceptor system (1A, D′-D-A-D-D′) shows weak AIE or ACQ behavior. The representative molecule 2TT-2BBTD (compound **10**) from the 2A system exhibits the highest molar absorptivity, intense NIR-II emission and superior AIE activity. Following nanoprecipitation with DSPE-mPEG2000, 2TT-2BBTD NPs were prepared (Figure 9B), demonstrating excellent colloidal stability, a spherical morphology with an average size of ~67 nm (Figure 9C), and strong NIR absorption/emission peaking at 799/1065 nm (Figure 9D). These NPs also show efficient ROS generation (Figure 9E) and high photothermal conversion efficiency (41.7%) under 808 nm laser irradiation (Figure 9F). In vivo, 2TT-2BBTD NPs enable high-resolution NIR-II FLI of blood vessels and lymph nodes (Figure 9G), along with clear PAI and PTI of orthotopic 4T1 breast tumors. The 2TT-2BBTD NPs accumulate efficiently in tumors, permitting fluorescence–photoacoustic–photothermal trimodal imaging-guided therapy. Upon laser irradiation, the combined PDT and PTT led to complete tumor eradication without recurrence (Figure 9H). Throughout the treatment, no significant body weight loss or organ damage is observed, confirming excellent biocompatibility (Figure 9I). This work thus validates that the “more is better” dual-acceptor approach not only boosts AIE and light-harvesting but also integrates NIR-II FLI, PAI, PTT, and PDT into a single, high-performance theranostic platform for precise cancer diagnosis and synergistic treatment.

Compounds **7** to **10** possess unique molecular structures and design strategies for multimodal phototherapy diagnosis. Compound **7** adopts a D-A-D structure, with the aniline-based benzothiophene donor carrying a large volume 2,4,4-trimethylpentyl group, achieving multi-dimensional donor engineering and balancing radiation attenuation and non-radiation attenuation. Compound **8**, with the TPE donor and benzodithiophene acceptor connected; it is also integrated into a mesoporous Prussian blue nanocarrier, where PB acts as a nano-catalyst for oxygen generation to enhance photodynamic therapy. Compound **9** has a D-π-A-π-D structure, with the tetrathiophene-based triphenylamine donor, EDOT π bridge, and selenium-substituted benzodithiophene acceptor components, achieving the first AIE small molecule that can be excited in the NIR-II region (1064 nm). Compound **10** adopts a dual receptor engineering strategy: two electron-deficient BBTD units are directly connected to form a highly twisted main chain structure, thereby achieving excellent AIE activity, enhanced molar absorptivity, and intense NIR-II band emission. In summary, these compounds differ in the main chain topological structure (D-A-D and D-π-A-π-D and A-A), donor/acceptor complexity, the use of nanocarriers, and the degree of receptor distortion.

#### 2.2.2. Thiadiazoloquinoxaline

**Scheme 4:**
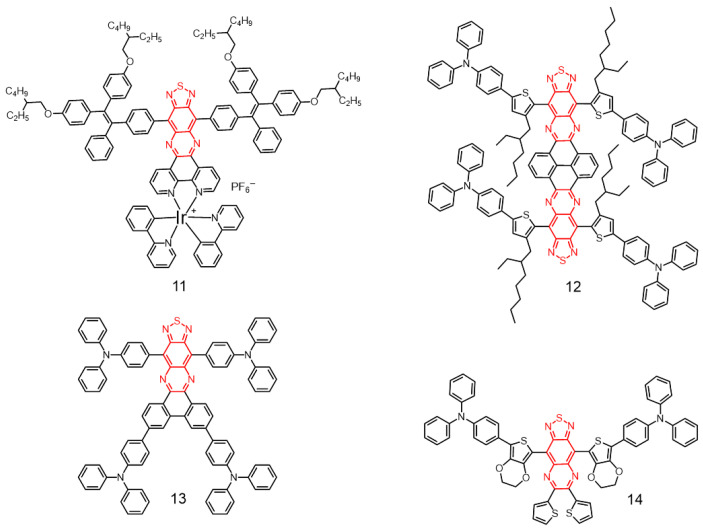
Molecular structures of D-A-D structured AIE compounds based on thiadiazoloquinoxaline for NIR-II fluorescence imaging, photoacoustic imaging, and applications in PTT and PDT.

In the design of NIR-II AIEgens for multimodal phototheranostics, the thiadiazoloquinoxaline moiety emerges as a quintessential and powerful electron-acceptor core (Scheme 4). Its planar, electron-deficient structure delivers a low-lying LUMO, a prerequisite for achieving a narrow energy gap to access the NIR-II spectral window for deep-tissue imaging. Strategically fused with donors like triphenylamine, it fosters a strong ICT effect, enabling finely tunable absorption and emission profiles. More importantly, within the twisted D-A-D framework, it critically aids in balancing excited-state energy dissipation pathways: restricting molecular motion to activate AIE, facilitating efficient intersystem crossing for type-I ROS generation, and enhancing non-radiative decay for superior photothermal conversion. Consequently, thiadiazoloquinoxaline is instrumental in constructing single-molecule entities capable of concurrent NIR-II FLI/PAI, and photothermal imaging, guided PDT and PTT. This structural motif underpins the development of advanced, stable, and biocompatible “all-in-one” phototheranostic platforms.

**Figure 10:**
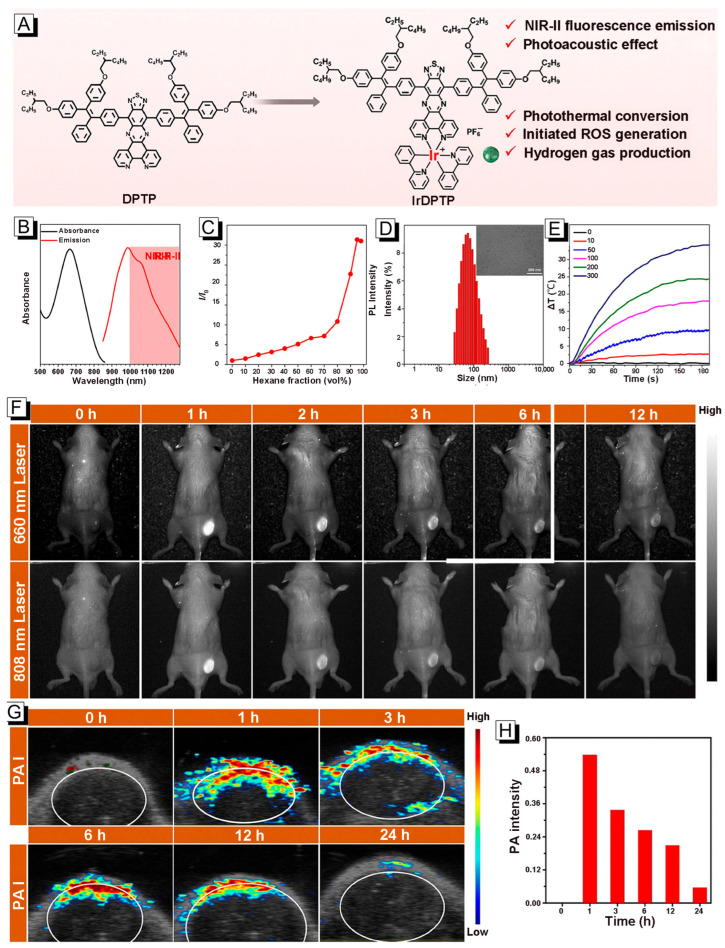
(**A**) Structure of IrDPTP. (**B**) Absorption/emission of Ir@PPEG-MeEPO. (**C**) AIE behavior vs. hexane fraction. (**D**) Size/TEM of NPs. (**E**) Photothermal heating at different concentrations. (**F**) In vivo NIR-II FLI post-injection. (**G**) In vivo PA images. (**H**) Quantified PA intensity at the tumor. Reprinted with permission from ref. [58]. © Springer Nature (2024).

The integrated nanomedicine Ir@PPEG-MeEPO combines an AIE-active Ir(III) complex (IrDPTP) (compound **11**) with a ^1^O_2_-charged amphiphilic polymer to enable a multi-functional theranostic platform [58]. The design leverages a D-A-D structured ligand to achieve NIR-II emission (Figure 10A), efficient ROS generation, and hydrogen production under NIR irradiation. Upon encapsulation, the resulting nanoparticles exhibit strong NIR-II fluorescence (peak at 1058 nm, Figure 10B), a large Stokes shift, and significant photothermal conversion efficiency, with temperature increases up to 34.3 °C under laser exposure (Figure 10E). The distinct AIE characteristic of the Ir(III) complex IrDPTP were demonstrates in Figure 10C, where its fluorescence intensity significantly increases in aggregated states formed in a poor solvent. This AIE property is pivotal, as it not only ensures intense and stable NIR-II emission for high-sensitivity fluorescence imaging but also contributes to efficient non-radiative energy dissipation, thereby enhancing the photothermal conversion capability and facilitating ROS generation for effective PDT. The nanoparticles demonstrate excellent stability in physiological conditions and a hydrodynamic size of ~68 nm (Figure 10D). Moreover, Ir@PPEG-MeEPO enables controlled release of ^1^O_2_ via photothermal activation of endoperoxide units, overcoming hypoxia limitations. In vivo studies reveal rapid tumor accumulation and retention, as shown by NIR-II FLI with peak signals at 1 h post-injection (Figure 10F). Concurrent PAI confirms deep-tissue penetration and real-time visualization of tumor contours, with PA intensity peaking similarly at 1 h (Figure 10G,H). This multimodal imaging capability (NIR-II FLI, PAI, and PTI) guides synergistic trimodal therapy combining PDT, PTT, and hydrogen therapy. The system demonstrates potent cytotoxicity under both normoxic and hypoxic conditions, with high phototoxicity indices, and achieves complete tumor eradication in vivo through combined PDT/PTT/hydrogen actions. Thus, Ir@PPEG-MeEPO represents a pioneering AIE-driven nanoplatform for sextuple theranostics, integrating NIR-II FLI-PAI-PTI trimodal imaging with PDT-PTT–hydrogen trimodal therapy for effective and precise cancer treatment.

**Figure 11:**
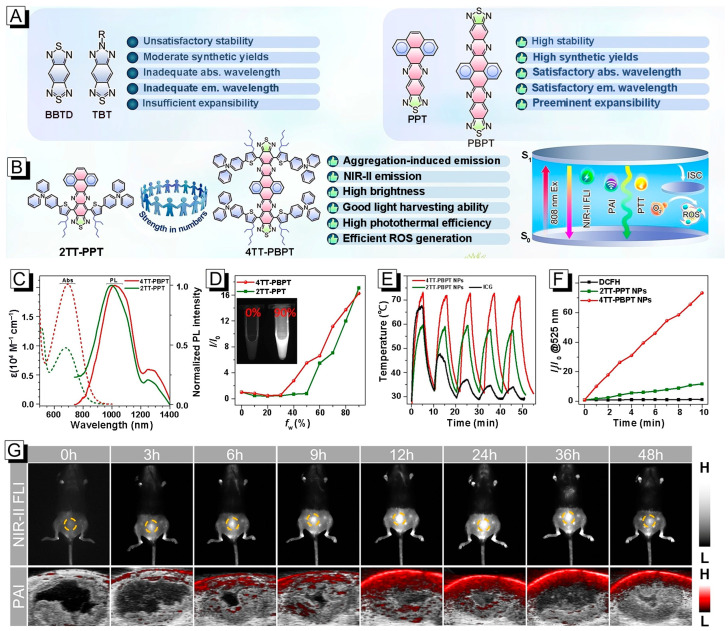
(**A**) Comparison of conventional vs. NHA acceptors. (**B**) Molecular design and π-extension strategy. (**C**) Absorption/PL of 2TT-PPT and 4TT-PBPT. (**D**) AIE behavior in THF/water. (**E**) Photothermal cycling vs. ICG. (**F**) ROS generation under 808 nm. (**G**) In vivo NIR-II FLI and PAI in orthotopic bladder tumor. Reprinted with permission from ref. [59]. © John Wiley and Sons (2025).

In 2025, You et al. reported a study on the rational design and synthesis of two novel N-heteroacenes (NHAs)-based NIR-II luminogens, 2TT-PPT and 4TT-PBPT (compound **12**), for multimodal imaging and synergistic therapy in orthotopic bladder cancer as one-for-all phototheranostic agents [59]. As illustrated in Figure 11A,B, the molecular structures are based on a D-A-D architecture, employing pyrene-fused phenazothiadiazole (PPT) and bisphenazothiadiazole (PBPT) as strong electron acceptors, with triphenylamine-based thiophene units acting as donors and molecular rotors. The photophysical properties in solution were demonstrated in Figure 11C, where 4TT-PBPT shows a red-shifted absorption peak at ~700 nm and emission at ~1026 nm compared to 2TT-PPT (~676 nm absorption, ~996 nm emission), indicating enhanced intramolecular charge transfer and stronger electron-withdrawing ability due to the extended π-conjugation. The AIE characteristics of both compounds (Figure 11D), with fluorescence intensity significantly increasing in THF/water mixtures at high water fractions, are crucial for preventing ACQ in the aggregate state. The photothermal stability of the prepared nanoparticles is validated in Figure 11E, where 4TT-PBPT NPs exhibit stable temperature changes over multiple lasers on/off cycles, outperforming the clinically used indocyanine green. The superior ROS generation capability of 4TT-PBPT NPs under 808 nm laser irradiation is shown in Figure 11F, which is essential for effective PDT. In vivo studies in an orthotopic bladder cancer model revealed that 4TT-PBPT NPs enabled high-contrast NIR-II FLI and PAI, with signal intensity peaking at 12 h post-injection (Figure 11G), providing optimal guidance for therapeutic intervention. The therapeutic efficacy was further validated by significant tumor weight reduction in the 4TT-PBPT NPs + laser group, while no notable body weight loss was observed, underscoring both potent anti-tumor activity and excellent biocompatibility. This work highlights the successful integration of NIR-II FLI, PAI, PTT, and PDT within a single AIE-active nanoplatform, offering a promising one-for-all strategy for precise imaging-guided synergistic treatment of orthotopic bladder cancer. Subsequently, Xiang et al. reported compound **13**, a D-A-D structured AIE photosensitizer that integrates FLI, PAI, PTT, and hypoxia-tolerant PDT into a single agent engineered for synergistic tumor theranostics [60].

**Figure 12:**
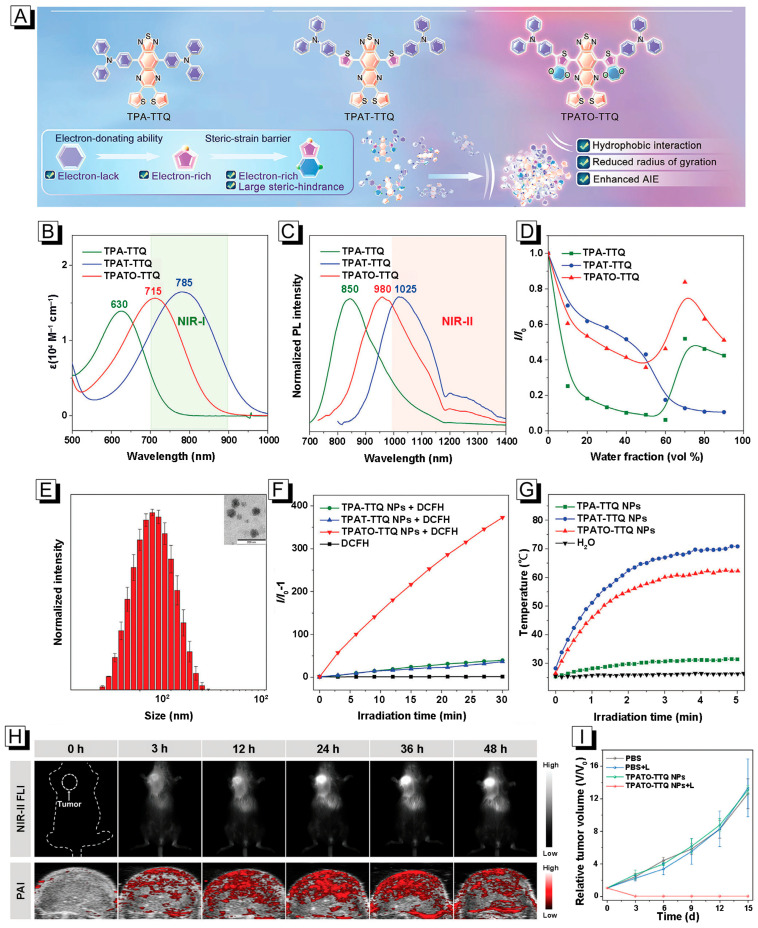
(**A**) Design of TPA-TTQ, TPAT-TTQ, TPATO-TTQ. (**B**) Absorption spectra. (**C**) Normalized PL in THF. (**D**) AIE behavior in THF/water. (**E**) Size/TEM of TPATO-TTQ NPs. (**F**) ROS generation. (**G**) Photothermal heating. (**H**) In vivo NIR-II FLI and PAI. (**I**) Tumor growth curves. Reprinted with permission from ref. [61]. © John Wiley and Sons (2025).

In 2025, Zhu et al. reported a study on the design, synthesis, and multimodal phototheranostic application of a novel NIR-II AIEgen, TPATO-TTQ (compound **14**), which integrates FLI, PAI, PTT, and PDT within a single molecular platform [61]. As illustrated in Figure 12A, the molecular engineering strategy involves tuning the π-bridge structure within a D-A-D framework to achieve balanced excited-state energy dissipation pathways. The molecular design of TPATO-TTQ with a sterically hindered π-bridge is to balance excited-state energy dissipation. The strong TTQ acceptor and bulky dihydrothienodioxine bridge restrict planarization and enhance molecular motion in aggregates. This promotes efficient NIR-II fluorescence, high photothermal conversion (η = 41.57%), and type-I ROS generation, enabling integrated FLI, PAI, PTT, and PDT within a single AIEgen system. Photophysical characterizations (Figure 12B,C) reveal that TPATO-TTQ exhibits strong NIR-II emission peaking at 980 nm in solution and demonstrates a significant AIE effect, with fluorescence enhancement in aqueous aggregates (Figure 12D). The AIEgen was formulated into nanoparticles with uniform size (about 100 nm by DLS, about 70 nm by TEM) and excellent colloidal stability (Figure 12E). Upon 808 nm laser irradiation, TPATO-TTQ NPs exhibit efficient photothermal conversion, reaching temperatures up to 62 °C with a high photothermal conversion efficiency of 41.57% (Figure 12G). Moreover, they demonstrate robust ROS generation capability, particularly through a type-I mechanism, as evidenced by a dramatic increase in DCFH fluorescence (Figure 12F). In vivo evaluations using an orthotopic 4T1 breast cancer model show that TPATO-TTQ NPs enable high-contrast NIR-II FLI and PAI, with optimal tumor accumulation observed at 36 h post-injection (Figure 12H). Guided by this trimodal imaging (FLI/PAI/PTI), synergistic PDT/PTT treatment led to complete tumor ablation without recurrence over 15 days, while control groups exhibited rapid tumor progression (Figure 12I). The work highlights TPATO-TTQ as a versatile AIE-based agent for precision cancer theranostics, combining deep-tissue imaging with potent phototherapeutic effects in a single-component system.

Compounds **11** to **14** correspond respectively to four NIR-II region diagnostic agents, each of which has a unique molecular structure and design strategy. Compound **11** is an iridium (III) complex containing a TPE unit. The large TPE rotor endows it with AIE properties, while the heavy atom effect promotes effective intermolecular crossing. Encapsulated in PPEG-MeEPO, this polymer can release ^1^O_2_ through thermal triggering, thereby overcoming the tumor hypoxic condition. Compound **12** adopts an N-heterocyclic ring receptor with four triphenylamine donors as the main structure. This design embodies the concept of “quantity advantage”. Its extended π conjugation structure and multiple donor arms enhance the D-A interaction, thereby achieving a high molar absorption coefficient, NIR-II emission, excellent photothermal conversion performance, and balanced multimodal diagnosis and treatment performance. Compound **13** is based on thiazolequinoline receptors and is formed by additional rings. Introducing a triphenylamine rotor forms a J-aggregate and achieves pure type-I sensitization without generating ^1^O_2_, thus being very suitable for low oxygen tumors. It also has a high photothermal conversion efficiency and NIR-II region fluorescence properties. Compound **14** has a D-π-A-π-D structure, where the π bridge (benzene, thiophene, or dihydrothiophene diketone) and donors have undergone systematic adjustments. Quantum chemical calculations and molecular dynamics analysis reveal how the internal molecular motion balances the radiative and non-radiative decay pathways, thereby achieving bright emission in the NIR-II region and high photothermal efficiency.

#### 2.2.3. Indanone-Condensed Thiadiazolo[3,4-*g*]quinoxaline (ITQ)

**Scheme 5:**
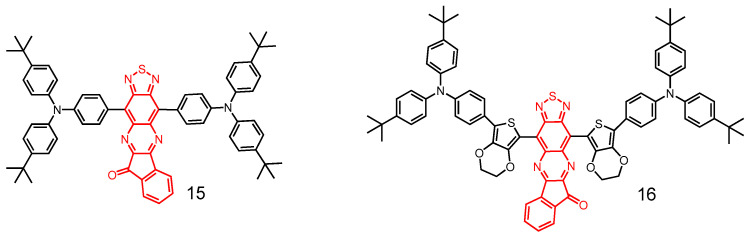
Molecular structures of D-A-D structured AIE compounds based on ITQ for NIR-II fluorescence imaging, photoacoustic imaging, and applications in PTT and PDT.

ITQ (indanone-condensed thiadiazolo[3,4-*g*]quinoxaline) serves as a powerful electron-accepting core in both studies, offering several key advantages for designing high-performance NIR-II AIEgens (Scheme 5). Firstly, its strong electron-deficient nature enhances intramolecular donor–acceptor (D-A) interaction, effectively narrowing the bandgap and enabling both absorption and emission to extend into the NIR-II region (1000–1700 nm). Secondly, the carbonyl group in the indanone moiety not only expands π-conjugation but also promotes spin–orbit coupling (SOC), facilitating efficient intersystem crossing (ISC) and boosting type-I ROS generation. Thirdly, ITQ’s rigid yet adaptable structure, when combined with twisted donor and *π*-bridges, helps suppress detrimental π-π stacking in aggregates, thereby enhancing AIE and providing loose molecular packing conducive to vigorous intramolecular motions. These features collectively allow ITQ-based molecules to achieve balanced radiative decay, efficient photothermal conversion, and potent type-I photodynamic activity, making them ideal for multimodal imaging-guided synergistic phototherapy.

**Figure 13:**
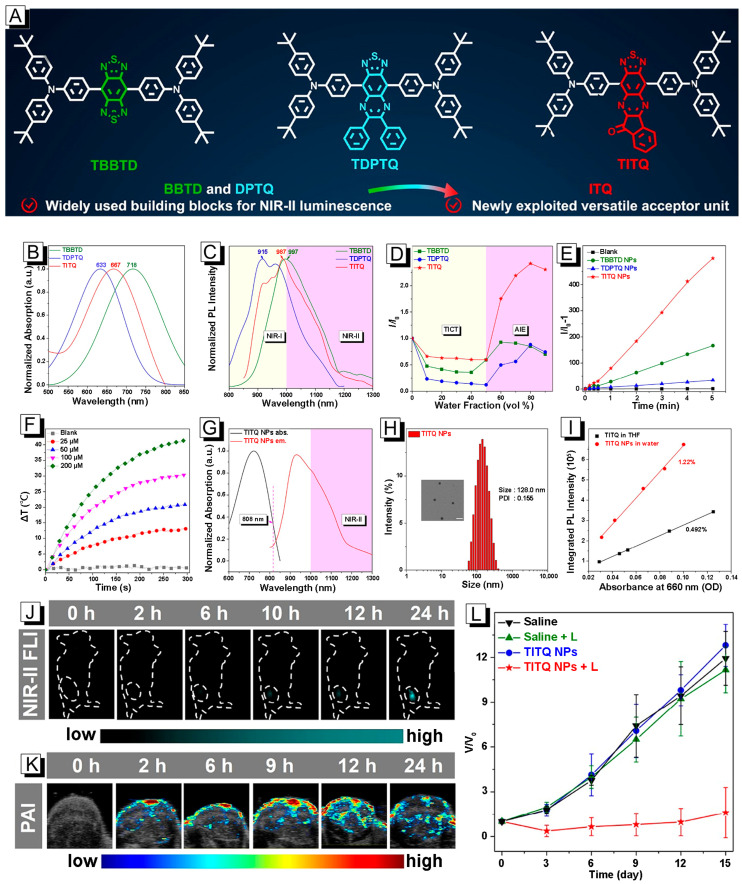
(**A**) Acceptor design strategies (TBBTD, TDPTQ, TITQ). (**B**,**C**) Absorption and PL in THF. (**D**) AIE behavior. (**E**) ROS generation of NPs. (**F**) Photothermal heating at varied concentrations. (**G**) Absorption/emission overlap of NPs. (**H**) DLS/TEM. (**I**) NIR-II emission vs. absorbance. (**J**) In vivo NIR-II FLI and (**K**) PA images. (**L**) Tumor growth kinetics. Reprinted with permission from ref. [62]. © Springer Nature (2024).

Li et al. reported a study on the development of a novel acceptor-engineered AIE photosensitizer, TITQ (compound **15**), tailored for NIR-II FLI and multimodal phototheranostics [62]. As illustrated in Figure 13A, the molecular design incorporates a newly developed indanone-condensed thiadiazoloquinoxaline (ITQ) acceptor, which endows TITQ with a highly twisted conformation, strong D-A interaction, and aggregation-induced NIR-II emission. The optical properties of TITQ in solution were demonstrated in Figure 13B,C, with broad absorption extending into the NIR region and bright NIR-II fluorescence with a large Stokes shift. The AIE behavior is confirmed in Figure 13D, where fluorescence intensity significantly enhances in aggregated states (high water fraction), attributed to restricted intramolecular motion. Moreover, TITQ exhibits robust ROS generation (Figure 13E) and efficient photothermal conversion (Figure 13F), enabling synergistic type-I photodynamic and photothermal therapy. When formulated into nanoparticles (TITQ NPs) via nanoprecipitation, the system shows excellent aqueous dispersibility and biocompatibility. Figure 13G reveals red-shifted absorption and slightly blue-shifted emission in the nanoparticle state, while DLS and TEM in Figure 13H confirm uniform spherical morphology with an average size of ~128 nm, ideal for EPR-mediated tumor targeting. The quantum yield of TITQ NPs is significantly improved compared to the solution state (Figure 13I), facilitating bright NIR-II fluorescence under various long-pass filters. In vivo studies on 4T1 tumor-bearing mice (Figure 13J,K) demonstrate that TITQ NPs efficiently accumulate at the tumor site over time, providing high-contrast NIR-II FLI and PAI with deep-tissue penetration and microscopic spatial resolution. The nanoprobe enables clear visualization of tumor vasculature and morphology, guided by its strong EPR-driven tumor retention. Furthermore, as shown in Figure 13L, TITQ NPs mediate potent tumor growth inhibition through combined photodynamic–photothermal therapy, underscoring their utility as an “all-in-one” phototheranostic platform. Together, these results highlight the successful integration of AIE-based NIR-II emission, multimodal imaging, and synergistic therapy in a single molecular system for advanced cancer phototheranostics.

**Figure 14:**
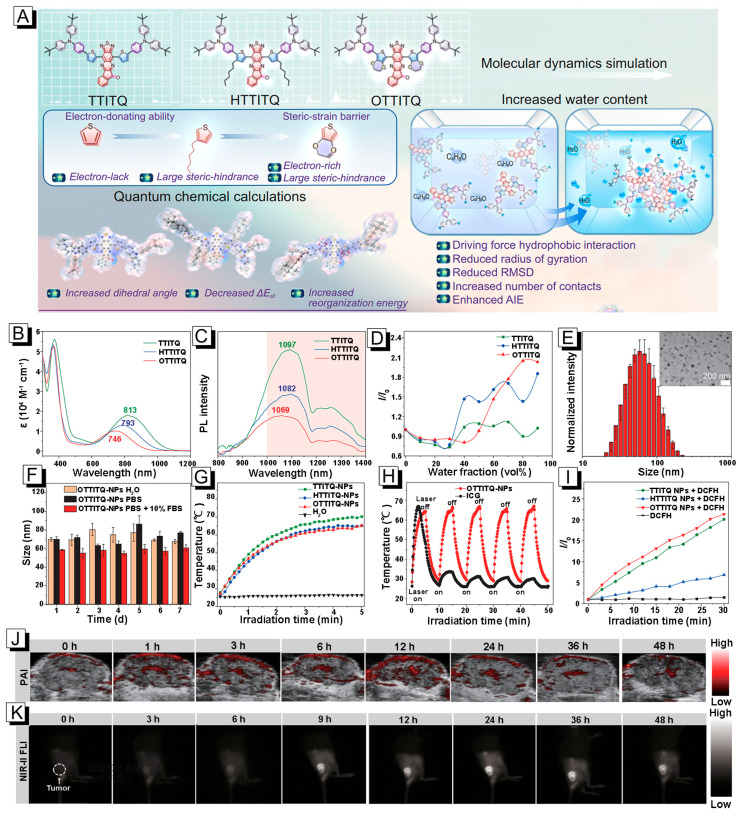
(**A**) Design and computational analysis of D-π-A-π-D AIEgens. (**B**,**C**) Absorption and PL in THF. (**D**) AIE behavior in THF/water. (**E**) Size/TEM of OTTITQ NPs. (**F**) Colloidal stability in different media. (**G**) Photothermal heating. (**H**) Photothermal cycling. (**I**) ROS generation. (**J**) In vivo NIR-II FLI and (**K**) PAI. Reprinted from ref. [63].

Zhu et al. reported a study on the design, synthesis, and application of NIR-II AIE luminogens (compound **16**) for advanced FLI and multimodal imaging-guided therapy [63]. As shown in Figure 14A, this molecule adopts a D-π-A-π-D structure, which includes a strong electron-accepting ITQ core and a molecular rotor, enabling the AIE effect in the NIR-II region. The photophysical properties analysis (Figure 14B,C) indicates that the optimized molecule OTTTQ has a strong absorption at 781 nm, and the spectral peak of the emitted light from the nanoparticle form is located at 1069 nm in the NIR-II region. Its spectral position has red-shifted compared to the solution state, confirming the effective AIE behavior. Figure 14D further demonstrates the AIE characteristics, where in a THF/water mixture, the fluorescence intensity increases with the increase in water content due to the effect of RIM. OTTTQ NPs exhibit excellent monodispersity (Figure 14E) and have good stability in physiological media (Figure 14F). These NPs also show significant photothermal conversion (Figure 14G,H) and efficient ROS generation (Figure 14I) under 808 nm laser irradiation, which are essential for combined PTT and PDT. In in vivo experiments, OTTTQ NPs were able to clearly achieve NIR-II band fluorescence imaging of mouse bladder tumors (Figure 14J), with the signal reaching its peak 24 h after injection and persisting for more than 36 h, indicating effective accumulation and retention of the tumors. Meanwhile, Figure 14K provided complementary structural visualization of the tumor with PAI, where the signal increased over time and was correlated with the accumulation of nanoparticles. In summary, these imaging methods achieved precise tumor depiction through AIE capabilities and provided guidance for subsequent photoligated therapies, highlighting the potential of NIR-II band AIE fluorophores in the comprehensive diagnosis and treatment platform for cancer.

#### 2.2.4. Naphtho[2,3-*c*][1,2,5]selenadiazole and Pyrazine-Based Planar Electronic Acceptor

**Scheme 6:**
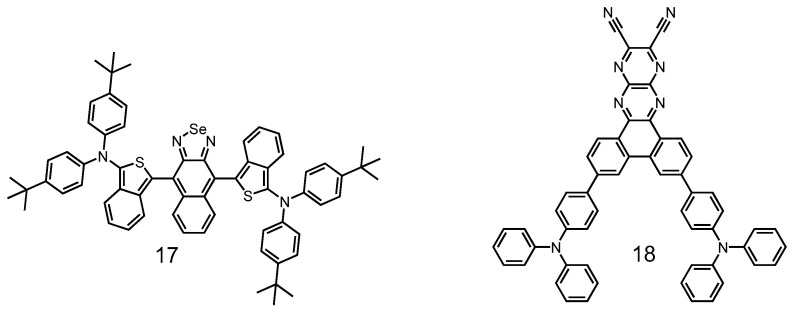
Molecular structures of D-A-D structured AIE compounds based on Naphtho[2,3-*c*][1,2,5]selenadiazole and pyrazine-based planar electronic acceptor for NIR-II fluorescence imaging, photoacoustic imaging, and applications in PTT and PDT.

The electron acceptor Naphtho[2,3-c][1,2,5]selenadiazole serves as a key structural component in the designed NIR-II AIEgens (Scheme 6). Its heavy-atom selenium promotes efficient intersystem crossing, enhancing ROS generation for photodynamic therapy, while its strong electron-withdrawing character narrows the molecular bandgap and red shifts emission into the NIR-II region. This dual functionality enables deep-tissue penetration for imaging and combined phototherapy, making it a pivotal building block for integrated multimodal phototheranostic agents.

**Figure 15:**
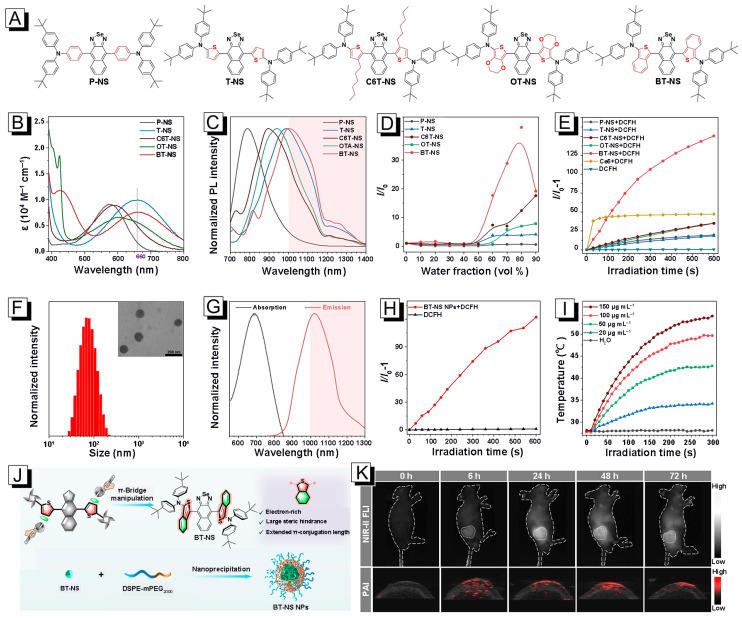
(**A**) Structures of P-NS, T-NS, C6T-NS, OT-NS, BT-NS. (**B**) Absorption and (**C**) PL in THF. (**D**) AIE behavior. (**E**) ROS generation. (**F**) Size/TEM of BT-NS NPs. (**G**) Absorption/emission in water. (**H**) Photothermal heating. (**I**) PCE calculation. (**J**) Schematic of π-bridge engineering. (**K**) In vivo NIR-II FLI and PAI. Reprinted with permission from ref. [64]. © John Wiley and Sons (2024).

The design and application of NIR-II AIEgens for FLI of vasculature and tumors are systematically demonstrated through strategic π-bridge engineering (compound **17**) [64]. As illustrated in Figure 15A,J, a series of AIEgens was constructed using different π-bridges, including phenyl, thiophene, ortho-alkylated thiophene, 3,4-ethylenedioxythiophene, and benzo[c]thiophene, to study their photophysical and theranostic behaviors. Among them, the benzo[c]thiophene-based AIEgen (BT-NS) exhibited the most favorable properties for NIR-II imaging. The absorption spectra of these AIEgens (Figure 15B) indicate that BT-NS possesses a strong absorption peak around 662 nm, which corresponds well to the 660 nm laser excitation wavelength. Moreover, BT-NS shows emission extending into the NIR-II region beyond 1000 nm, with a maximum at 1003 nm (Figure 15C), thereby enabling deep-tissue fluorescence imaging with reduced scattering and minimal autofluorescence. The AIE characteristics are confirmed in Figure 15D, where fluorescence intensity increases significantly in aggregate state (THF/water mixtures), confirming the aggregation-induced emission behavior essential for bright imaging in biological environments. Furthermore, the efficient ROS generation capability of BT-NS under laser irradiation is shown in Figure 15E,H, supporting its dual role in imaging and therapy. For biomedical applications, BT-NS was encapsulated into nanoparticles using DSPE-mPEG_2000_ (Figure 15J). Figure 15F shows that BT-NS NPs are spherical with an average diameter of ~67 nm, ideal for passive tumor targeting via the EPR effect. The optical properties are retained in aqueous dispersion, as seen in Figure 15G, with absorption and emission peaks red-shifted to 693 nm and 1020 nm, respectively. The photothermal performance and ROS generation capability of BT-NS NPs are highlighted in Figure 15I, where BT-NS NPs show concentration-dependent temperature rise and a high photothermal conversion efficiency of ~41.8%, supporting their use in PTI and therapy. In vivo NIR-II FLI (Figure 15K) reveals gradual accumulation of BT-NS NPs at the tumor site over time, with peak intensity at 24 h post-injection, demonstrating effective tumor targeting and retention suitable for vascular and tumor imaging. Together, these results underscore the potential of π-bridge-tailored NIR-II AIEgens like BT-NS for high-contrast, deep-tissue fluorescence imaging of vasculature and tumors, complemented by multimodal imaging and therapeutic capabilities.

**Figure 16:**
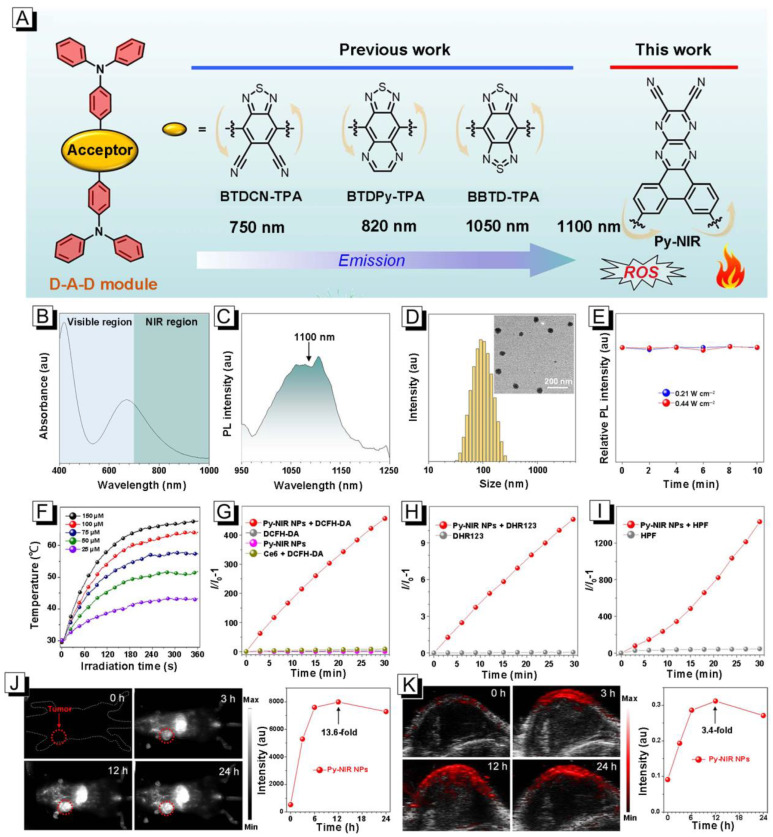
(**A**) Structural evolution from BTD to pyrazine-based acceptor. (**B**,**C**) Absorption/PL of Py-NIR NPs. (**D**) DLS/TEM. (**E**) Photostability under laser. (**F**) Photothermal heating. (**G**–**I**) ROS detection with different probes. (**J**) In vivo NIR-II FLI time series. (**K**) PAI and signal intensity plot. Reprinted from ref. [65].

Chen et al. published a study on the development of NIR-II band fluorescent agents with AIE properties, highlighting the significant progress made in deep-tissue vascular and tumor imaging (compound **18**) [65]. As illustrated in Figure 16A, the planar pyrazine-based electronic acceptor core enables the construction of a D-A-D AIEgen. The optical properties are confirmed in Figure 16B,C, where Py-NIR NPs show distinct absorption peaks and a strong NIR-II photoluminescence peak. The NPs exhibit spherical morphology (Figure 16D), with remarkable photostability (Figure 16E). Furthermore, Py-NIR NPs demonstrate efficient photothermal conversion and type-I photosensitization (Figure 16G–I). In vivo NIR-II fluorescence imaging (Figure 16J) shows clear tumor delineation peaking at 12 h post-injection. Additionally, the photothermal and photoacoustic imaging capabilities of Py-NIR NPs (Figure 16K) provide complementary spatial and functional information, enabling multimodal imaging-guided tumor ablation.

### 2.3. Design Strategies of A-D-A Type Molecules and Their Advantages in NIR-II FLI/PAI, PTT/PDT, and AIE Systems

The design of A-D-A type NIR-II molecules integrates a highly planar donor–acceptor core to achieve strong molar absorptivity and near-infrared absorption. A key strategy involves modifying the acceptor unit with bulky substituents to induce a reversely staggered molecular packing. Furthermore, the incorporation of flexible, rotational moieties within the structure acts as efficient molecular rotors, facilitating non-radiative decay and significantly boosting the photothermal effect.

**Figure 17:**
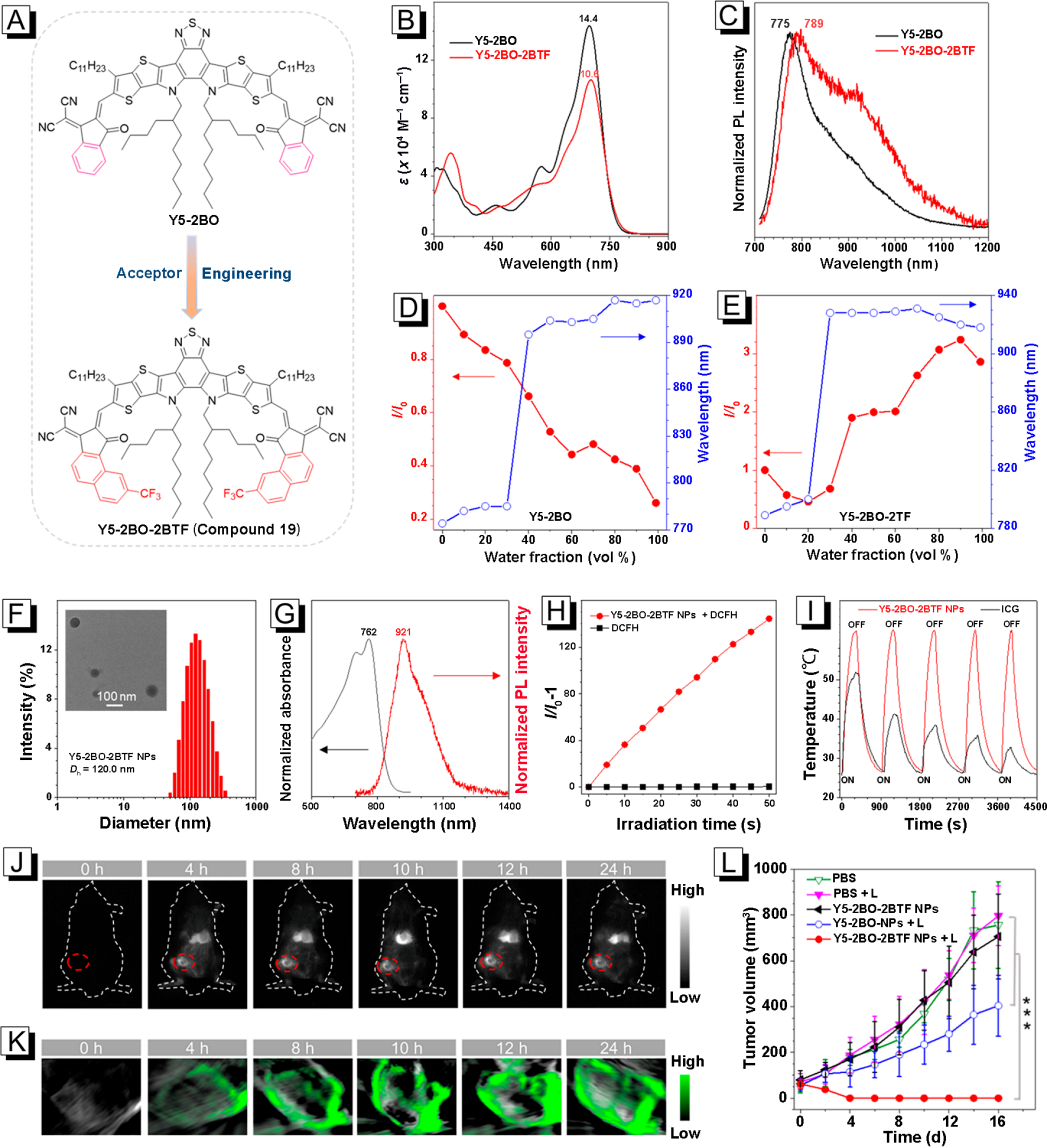
(**A**) Structures of Y5-2BO and Y5-2BO-2BTF. (**B**,**C**) Absorption and PL in THF. (**D**,**E**) AIE behavior in THF/water. (**F**) Size/TEM of NPs. (**G**) Absorption/emission in water. (**H**) ROS generation. (**I**) Photothermal cycling vs. ICG. (**J**) In vivo NIR-II FLI and (**K**) PAI. (**L**) Tumor growth curves (*** *p* < 0.001. Student’s *t*-test). Reprinted with permission from ref. [66]. © American Chemical Society (2025).

Yang et al. reported Y5-2BO-2BTF (compound **19**), designed for multimodal phototheranostics [66]. The chemical structures of the planar control molecule Y5-2BO and the modified Y5-2BO-2BTF are shown in Figure 17A, where meta-CF_3_-substituted naphthyl groups are introduced to create steric hindrance. The absorption spectra (Figure 17B) in THF solution reveal strong NIR absorption peaks around 700 nm with exceptionally high molar absorptivity (*ε*) of 1.06 × 10^5^ M^−1^ cm^−1^ for Y5-2BO-2BTF. The photoluminescence spectra (Figure 17C) demonstrate a red-shifted emission maximum at 789 nm for Y5-2BO-2BTF compared to Y5-2BO (775 nm), indicating enhanced intramolecular charge transfer. Figure 17D,E illustrate the AIE behavior of Y5-2BO-2BTF in THF/water mixtures, where fluorescence intensity significantly recovers and increases at high water fractions, confirming the transformation from ACQ to AIE character. For biological applications, Y5-2BO-2BTF was formulated into nanoparticles. SLD and TEM (Figure 17F) confirm the formation of spherical NPs (~120 nm). The NPs exhibit strong absorption at 762 nm and NIR-II emission at 921 nm (Figure 17G), suitable for deep-tissue imaging. Figure 17H demonstrates the efficient generation of ROS, particularly type-I ROS (•OH and O_2_^−^•), under laser irradiation, which is crucial for PDT even in hypoxic tumor microenvironments. The photothermal performance is outstanding, as shown in Figure 17I, where Y5-2BO-2BTF NPs maintain stable and superior photothermal heating over multiple laser on/off cycles, achieving a high photothermal conversion efficiency (PCE) of 77.8%, compared to the rapid degradation of the reference ICG. The in vivo efficacy of this multimodal platform is validated in orthotopic breast tumor models. Figure 17J shows clear, time-dependent NIR-II FLI of tumors after intravenous injection of Y5-2BO-2BTF NPs, with optimal accumulation at 12 h. This high-contrast FLI is complemented by strong photoacoustic signals (Figure 17K), providing complementary anatomical and functional information for precise tumor localization. Ultimately, this imaging guidance enables highly effective combination therapy. As summarized in Figure 17L, mice treated with Y5-2BO-2BTF NPs followed by 808 nm laser irradiation exhibit complete tumor eradication and inhibition of metastasis, outperforming all control groups. This superior outcome stems from the synergistic “PTT/PDT” effect powered by the AIEgen’s high ε for efficient photon harvesting, its loose aggregate packing and active -CF_3_ rotors for ultrahigh PTT efficiency, and its ability to generate toxic type-I ROS for hypoxia-tolerant PDT. Thus, this work establishes a novel paradigm of “motion and stillness” for engineering single-molecule-based AIEgens that integrate high-efficiency NIR-II PAI, PTT, and PDT into a unified, high-precision cancer theranostic platform.

The key photophysical and therapeutic parameters of the above AIE-based phototheranostic agents are summarized in Table 1. It should be noted that currently, there is a lack of a structured quantitative measurement method to directly compare the compounds, and the methodological differences in different studies (including differences in excitation wavelength, laser power density, administration route, and tumor models) limit the direct comparability of parameters such as PCE, ROS generation, and in vivo efficacy. However, some trends can still be observed: most compounds show a red shift (e.g., compound **1**: 860 to 865 nm; compound **4**: ~1000 to ~1060 nm), which is consistent with the AIE mechanism; while a few compounds show a slight blue shift (e.g., compound **7**: 1100 to 1063 nm). compound **19** achieved the highest PCE (77.8%), followed by compound **12** (73.8%) and compound **9** (56.6%). Almost all compounds exhibit NIR-II emission (>1000 nm) in the nanoparticle state, with excitation mainly at 808 nm, while a few compounds use 1064 nm to achieve deeper tissue penetration. Intravenous administration is the main administration method, and multimodal imaging (NIR-II FLI/PAI/PTI) combined with PDT/PTT constitutes the core treatment strategy. Representative achievements include compound **19** (with the highest drug concentration, capable of completely eliminating tumors and inhibiting metastasis), compound **12** (the first NIR-II AIE molecule for in situ bladder cancer), and compound **9** (the first small molecule with NIR-II AIE properties excited at 1064 nm). Despite the methodological differences, this summary provides a useful overview of the molecular design strategies and therapeutic effects.

### 2.4. Computational Insights into Structure/Property Relationships

Bandgap tuning and absorption/emission in the NIR-II band. The energy gap between the HOMO and the LUMO of the molecule was calculated using DFT to explain the red-shift phenomenon. For example, when designing compound **15**, introducing the benzothiophenone-type receptor reduced the energy gap from 1.79 eV to 1.64 eV, which was consistent with the red shift of the absorption wavelength from 633 nm to 718 nm. Similarly, for compound **9**, replacing sulfur in the receptor with selenium reduced the energy gap to 1.12 eV, enabling absorption at 915 nm and emission at 1240 nm. These calculations provide quantitative guidance for selecting receptor units to achieve the target NIR-II region.

AIE properties and intramolecular motion. In several studies (e.g., compounds **10**, **14**, **16**, **19**), the reorganization energy (λ) was analyzed using the MOMAP software package (1.0.0) to quantify the contribution of dihedral angle changes to non-radiative decay. For YS-2BO-2BTF (compound **19**), the molecule showed a high λ value dominated by dihedral angle torsion, indicating active intramolecular motion in solution. In the aggregated state, the anti-intercalated stacking observed in the single crystal inhibited π-π stacking while retaining the rotation of the –CF_3_ group, which explained its excellent AIE properties and a photoelectric conversion efficiency of up to 77.8%. These analyses directly linked the molecular conformation to the AIE behavior.

Triplet state formation and ROS generation. The singlet–triplet energy splitting (ΔE_ST_) and spin–orbit coupling (SOC) constants derived from the TD-DFT method were used to explain the efficiency of ROS generation. For PTQ-TPA3 (compound **13**), the geometric structure produced a ΔEST of 0.08 eV and enhanced SOC, thereby promoting efficient type-I ROS generation under low oxygen conditions. Insights from these computational aspects guided the design of oxygen-tolerant photosensitizers.

## 3. Challenges and Perspectives

This review systematically summarizes the rational design principles and significant progress of AIEgens in the NIR-II window, which are used in integrated optical therapy and diagnosis technologies. Through strategic molecular engineering with strong D-A interaction, prolonging the π conjugation, and introducing twisted or macromolecular groups, these AIE active molecules achieve a red shift of absorption/emission to the NIR-II region, enhance the brightness of the aggregated state, and achieve a balance between radiative and non-radiative decay pathways. This enables seamless integration of multiple functions within a single platform: high-contrast NIR-II FLI for precise tumor delineation, efficient PTT/PDT for ablation. The resulting nanodelivery systems exhibit excellent stability, biocompatibility, and tumor targeting ability, achieved through enhanced permeability and retention effects.

Challenges: Biocompatibility: Most of the literature usually concludes “excellent biocompatibility” based on short-term (e.g., 14 days) weight monitoring and H&E staining of major organs. However, we now clearly state that these observations do not guarantee long-term safety. We emphasize that before clinical translation, longer toxicity studies, pharmacokinetic analysis, and assessment of potential chronic inflammation or immune responses are necessary. Tumor eradication: Most literature reports are based on short observation windows (14 days), reporting “complete tumor eradication” or “no recurrence”. We must carefully point out that such findings should be interpreted with caution, as few assess long-term recurrence and metastasis potential. Most studies use immune-deficient mouse models, which cannot replicate the complexity of the immune system in immunocompetent hosts—this is a key factor in evaluating the efficacy of combined immunotherapy. Translational potential: Due to the gap between nanoparticle research and clinical needs, most studies achieve passive targeting through the endothelial–vascular response effect. However, this effect has high inter-patient and inter-tumor differences in humans, highlighting the necessity of adopting an active targeting strategy.

Perspectives: Apart from in vivo imaging and therapy, the application of NIR-II level spontaneous fluorescence enhancers in the study of intracellular metabolic processes in in vitro models remains an emerging but not yet fully explored field [67,68]. Compared with traditional fluorescent probes, NIR-II level spontaneous fluorescence enhancers have excellent photostability, extremely low background interference, and the ability to detect dynamic metabolic events in complex three-dimensional microenvironments in real time. With the development of technology, combining NIR-II type spontaneous fluorescence enhancers with advanced tissue and organ models, as well as high-content imaging platforms, is expected to bridge the gap between basic metabolic research and translational treatment development. Looking ahead, there are still some key challenges in applying NIR-II materials in clinical translation. First, the synthesis routes and purification processes need to be optimized to enable large-scale production. Second, comprehensive long-term biological safety assessments must be conducted. Third, radiation parameters such as laser wavelength, power density, irradiation time, and treatment frequency need to be standardized to ensure the consistency and reproducibility of the therapeutic effect. Fourth, active targeting ligands (antibodies, peptides, aptamers) can be used to reduce non-targeted effects and improve the therapeutic index. In terms of opportunities, the combination of NIR-II molecules with emerging therapies (such as immunotherapy and ferroptosis induction) has the potential for synergistic treatment. The development of activatable probes that can respond to signals from the tumor microenvironment (pH value, enzymes, etc.) enables on-demand, precise activation of imaging and treatment. Solving these challenges and seizing these opportunities will greatly promote the clinical application transformation of such nanomedicines.

## Figures and Tables

**Table 1:** Summary of AIE-based compounds with in vivo phototheranostic performance.

Compound	λ_abs_ (nm)	λ_em, sol._ (nm)	λ_em, NPs_ (nm)	PCE (%)	Excitation (Ex)	Ref.
Compound **1**	560 (NPs)	860 (EtOH)	865 (H_2_O)	40.5	635 nm	[41]
Compound **2**	664 (Solid)	N/A	992 (H_2_O)	46	660 nm	[23]
Compound **3**	~640 (film)	1022 (DMSO)	950 (H_2_O)	40.1	660 nm	[46]
Compound **4**	~770 (NPs)	~1000 (THF)	~1060 (H_2_O)	35	1064 nm	[47]
Compound **5**	568 (dots)	813 (DMSO/toluene)	N/A	39.9	660 nm	[48]
Compound **6**	677 (DMSO)	938 (PhMe)	N/A	37.8	671 nm	[49]
Compound **7**	805 (NPs)	1100 (THF)	1063 (H_2_O)	28.8	808 nm	[56]
Compound **8**	708 (NPs)	N/A	982 (H_2_O)	53.1	730 nm	[57]
Compound **9**	915 (CHCl_3_)	1240 (CHCl_3_)	~1250 (H_2_O)	56.6	1064 nm	[51]
Compound **10**	773 (THF)	1004 (THF)	1065 (H_2_O)	41.7	808 nm	[52]
Compound **11**	663 (NPs)	N/A	1058 (H_2_O)	N/A	660 nm	[58]
Compound **12**	700 (THF)	1026 (THF)	N/A	73.8	808 nm	[59]
Compound **13**	735 (NPs)	955 (THF)	955 (H_2_O)	39.45	808 nm	[60]
Compound **14**	730 (NPs)	980 (THF)	1000 (H_2_O)	41.57	808 nm	[61]
Compound **15**	718 (THF)	915 (THF)	N/A	23.6	808 nm	[62]
Compound **16**	746 (THF)	1069 (THF)	1084 (H_2_O)	35.63	808 nm	[63]
Compound **17**	693 (NPs)	1003 (THF)	1020 (H_2_O)	41.8	660 nm	[64]
Compound **18**	670 (NPs)	N/A	1100 (H_2_O)	56.1	808 nm	[65]
Compound **19**	762 (NPs)	789 (THF)	921 (H_2_O)	77.8	808 nm	[66]

## Data Availability

No new data were created or analyzed in this study.

## Abbreviations

The following abbreviations are used in this manuscript:

NIR-II	second near-infrared
AIE	aggregation-induced emission
AIEgens	aggregation-induced emission luminogens
FLI	fluorescence imaging
PAI	photoacoustic imaging
PTT	photothermal therapy
PDT	photodynamic therapy
ACQ	aggregation-caused quenching
RIM	restriction of intramolecular motion
ICT	intramolecular charge transfer
D-A	Donor-acceptor
D-A-D	Donor-acceptor-donor
A-D-A	Acceptor-donor–acceptor
NPs	nanoparticles
EPR	enhanced permeability and retention
TPA	triphenylamine
TEM	transmission electron microscopy
DLS	dynamic light scattering
